# Membrane-Targeting Triphenylphosphonium Functionalized Ciprofloxacin for Methicillin-Resistant *Staphylococcus aureus* (MRSA)

**DOI:** 10.3390/antibiotics9110758

**Published:** 2020-10-30

**Authors:** Sangrim Kang, Kyoung Sunwoo, Yuna Jung, Junho K. Hur, Ki-Ho Park, Jong Seung Kim, Dokyoung Kim

**Affiliations:** 1 Department of Anatomy and Neurobiology, College of Medicine, Kyung Hee University, Seoul 02447, Korea; sangrimk@gmail.com; 2 Department of Chemistry, Korea University, Seoul 02841, Korea; swk9028@naver.com; 3 Department of Biomedical Science, Graduate School, Kyung Hee University, Seoul 02447, Korea; jungpeng159@gmail.com; 4 Department of Genetics, College of Medicine, Hanyang University, Seoul 04763, Korea; juhur@hanyang.ac.kr; 5 Division of Infectious Diseases, Department of Internal Medicine, Kyung Hee University, Seoul 02447, Korea; 6 Center for Converging Humanities, Kyung Hee University, Seoul 02447, Korea; 7 Medical Research Center for Bioreaction to Reactive Oxygen Species and Biomedical Science Institute, School of Medicine, Graduate School, Kyung Hee University, Seoul 02447, Korea

**Keywords:** antibiotic conjugates, ciprofloxacin, multidrug resistance bacteria, triphenyl-phosphonium

## Abstract

Multidrug-resistant (MDR) bacteria have become a severe problem for public health. Developing new antibiotics for MDR bacteria is difficult, from inception to the clinically approved stage. Here, we have used a new approach, modification of an antibiotic, ciprofloxacin (CFX), with triphenylphosphonium (TPP, PPh_3_) moiety via ester- (CFX-ester-PPh_3_) and amide-coupling (CFX-amide-PPh_3_) to target bacterial membranes. In this study, we have evaluated the antibacterial activities of CFX and its derivatives against 16 species of bacteria, including MDR bacteria, using minimum inhibitory concentration (MIC) assay, morphological monitoring, and expression of resistance-related genes. TPP-conjugated CFX, CFX-ester-PPh_3_, and CFX-amide-PPh_3_ showed significantly improved antibacterial activity against Gram-positive bacteria, *Staphylococcus aureus*, including MDR *S. aureus* (methicillin-resistant *S. aureus* (MRSA)) strains. The MRSA ST5 5016 strain showed high antibacterial activity, with MIC values of 11.12 µg/mL for CFX-ester-PPh_3_ and 2.78 µg/mL for CFX-amide-PPh_3_. The CFX derivatives inhibited biofilm formation in MRSA by more than 74.9% of CFX-amide-PPh_3_. In the sub-MIC, CFX derivatives induced significant morphological changes in MRSA, including irregular deformation and membrane disruption, accompanied by a decrease in the level of resistance-related gene expression. With these promising results, this method is very likely to combat MDR bacteria through a simple TPP moiety modification of known antibiotics, which can be readily prepared at clinical sites.

## 1. Introduction

Antimicrobial resistance is considered to be one of the greatest threats to human health worldwide. Widespread abuse and misuse of antibiotics has led to the emergence of antibiotic resistant bacteria [1,2]. Microbes resistant to multiple anti-microbials are known as multidrug-resistant (MDR) and cannot be easily killed using the current drugs. The MDR bacterial strains include the “ESKAPE” pathogens—*Enterococcus faecium*, *Staphylococcus aureus*, *Klebsiella pneumoniae*, *Acinetobacter baumannii*, *Pseudomonas aeruginosa*, and *Enterobacter* spp. The ESKAPE pathogens play a critical role in nosocomial infections and resist antimicrobial agents through drug resistance mechanisms [3]. As a result, patients infected with MDR bacteria are harder to treat, thereby increasing medical costs exorbitantly, in comparison to patients infected with bacteria more susceptible to drugs [4]. To date, many approaches have been proposed to overcome MDR bacteria, including ESKAPE pathogens, such as use of drug combination, drug modification, and drug delivery systems [5]. Many new antimicrobial agents, such as inhibitors of quorum sensing (QS) [6], antimicrobial peptides (AMPs) [7], and bacteriophages [8], have been suggested, and they are based on the target of the molecular mechanisms of MDR. In addition, various approaches based on inhibiting the formation of the negatively charged membrane bilayer, using cationic polymers and inhibitory integral proteins, similar to membrane bound multidrug efflux pumps, have been shown to be effective against both Gram-negative and Gram-positive MDR bacteria. However, these methods have proven to be difficult to develop into an approved clinical medicine [9,10].

**Figure 1:**
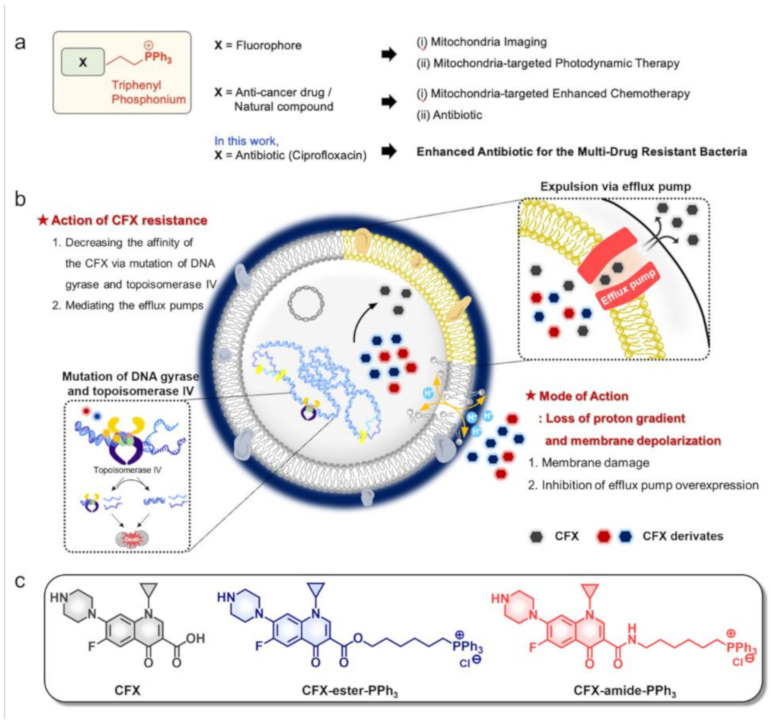
Schematic summary of the approach used and the motivation for this work. (**a**) Applications of triphenylphosphonium (TPP^+^, PPh_3_). In this work: X = antibiotic (ciprofloxacin) used to enhance the effect of antibiotics against multidrug-resistant (MDR) bacteria. (**b**) A schematic diagram showing the mechanism of drug-resistance in bacteria and the mode of action towards ciprofloxacin (CFX) and the functioning mechanism of CFX-PPh_3_ derivatives. (**c**) Chemical structure of CFX and its derivatives; CFX-ester-PPh_3_, CFX-amide-PPh_3_**.**

In this study, we disclosed a strategy to overcome problems associated with traditional approaches against MDR bacteria, which utilize (i) triphenylphosphonium (TPP, PPh_3_) moiety and (ii) ciprofloxacin (CFX). (i) TPP: Positively charged TPP has been widely used for targeting negatively charged sub-cellular organelles, such as mitochondria (Figure 1a). Mitochondria-targeted cell imaging and mitochondria-related biostudy, achieved by introducing TPP into the fluorophore, are widely used techniques. Moreover, tagging TPP to an anticancer drug has shown enhanced mitochondria-targeted chemo-therapeutic efficiency [11,12,13]. TPP-conjugated compounds have significant antibacterial activity against both Gram-negative and Gram-positive bacteria, due to the large hydrophobic surface area and delocalized charge distribution of TPP, which allows them to easily cross biological membranes [14]. (ii) CFX: CFX has been widely used for treating various bacterial infections, such as urinary tract infections, acute sinusitis, and chronic bacterial prostatitis [15]. CFX is a fluoroquinolone antibiotic that is effective against both Gram-negative and Gram-positive bacteria. CFX interferes with bacterial DNA replication and transcription by inhibiting bacterial DNA gyrase, topoisomerase II, and DNA topoisomerase IV. Traditionally, CFX has been used for treating severe infections caused by *S. aureus*. However, over a period of time, *S. aureus* and isolates of methicillin-resistant *S. aureus* (MRSA) have become resistant towards CFX and other fluoroquinolone antibiotics [16,17]. Two different mechanisms are known to lead to resistance to fluoroquinolones, including CFX, (i) by decreasing the affinity of CFX via mutations occurring in the quinolone-resistance-determining region of topoisomerase IV and DNA gyrase and (ii) by inhibiting the efflux pumps, which is less recognized (Figure 1b) [18].

Given the similarities between the mitochondrial and bacterial membranes, we have developed two derivatives of TPP-conjugated CFX, one has an ester bond (CFX-ester-PPh_3_) and the other has an amide bond (CFX-amide-PPh_3_) to increase the antibacterial activities of CFX by targeting the bacterial membrane (Figure 1c). We systematically examined the antimicrobial activities of TPP-conjugated CFX against 16 types of ESKAPE bacteria, including strains of MDR bacteria (Table 1). TPP-conjugated CFXs were found to be significantly effective against Gram-positive bacteria, *S. aureus* and MRSA by disrupting the cell membrane and inhibiting the multidrug efflux pump (Figure 1b). This study has successfully demonstrated the efficiency of the TPP-CFX hybridization approach for combating MDR bacteria.

## 2. Results and Discussion

### 2.1. Rational

One of the new strategies to overcome MDR bacteria is to develop membrane-active antibacterial agents. Such agents have an antibacterial action as follows: (i) to cause collapsing of the membrane architecture by interacting with a lipophilic moiety on the bacterial membrane, (ii) to make complex and/or localization into membrane-embedded proteins, and (iii) to alter the proton motive force (PMF) [19]. We focused on the two mechanisms of action, first is the alteration of PMF, which affects the operation system of proton-dependent multidrug efflux system, and the other is collapsing the bacterial membrane. We used the CFX as a traditional antibiotic in this study. As we described above, the resistance mechanism of CFX is that CFX binds to DNA gyrase and topoisomerase II/IV for inhibition of DNA replication and transcription. It appears to occur both by mutation of DNA gyrase and topoisomerase II/IV and by alteration of CFX permeation via the cell membrane. Thus, we designed TPP-conjugated CFX derivatives to target anionic lipids of the bacterial membrane (Figure 1b). It is known that the covalently linked TPP moiety enhances the lipophilic character of TPP-conjugated CFXs derivatives, and this strategy has been used to increase the solubility of drugs and imaging agents, and improve their bioactivity [12,20]. The TPP moiety was conjugated on the carboxylic acid moiety of CFX via esterification (for CFX-ester-PPh_3_) and amide coupling (for CFX-amide-PPh_3_) using triphenyl-phosphonium propyl alcohol, and triphenyl-phosphonium propyl amine, respectively. The derivatives were prepared using the protocol developed by our group (previous work: drug repositioning of antibiotic for anticancer) (Scheme S1 and S2 in Supplementary material) [21]. CFX-ester-PPh_3_ was prepared by using *t*-butyloxycarbonyl (Boc)-protected CFX and triphenyl-phosphonium propyl alcohol, with potassium carbonate (K_2_CO_3_) in *N*,*N*-dimethylformamide (DMF). The reaction mixture was stirred at 50 °C for overnight, then purified by column chromatography (yield: 82%). The deprotection of the Boc group was carried out in an acidic condition (yield: 90%). CFX-amide-PPh_3_ was prepared in a similar way by amide coupling, using the Boc-protected CFX and triphenyl-phosphonium propyl amine with 1-ethyl-3-(3-dimethylaminopropyl) carbodiimide (EDC) and 4-dimethylaminopyridine (DMAP) catalyst in DMF. The reaction mixture was stirred at room temperature overnight and purified by silica-gel column chromatography (yield: 78%). Thereafter, deprotection of the Boc group was carried out in an acidic condition (yield: 93%). The purity of the synthesized compounds was verified by proton/carbon nuclear magnetic resonance (^1^H/^13^C-NMR) and electrospray ionization mass spectrometry.

### 2.2. MIC Assay

Antibacterial activity of TPP-conjugated CFX derivatives was evaluated against 16 types of Gram-positive and Gram-negative bacterial strains, including MDR bacteria (Table 1). Non-derivatized CFX was used as a control to compare the activities of the CFX-PPh_3_ derivatives. The minimum inhibitory concentration (MIC) values of CFX for all types of strains were produced with reference to the Clinical and Laboratory Standards Institute (CLSI) recommended breakpoints (Table 2) [22]. MIC assays of CFX and CFX-PPh_3_ derivatives against bacteria strains (16 types) were performed using the broth micro-dilution method in a 96-well plate and represented in Figure S1 (Supplementary Material).

**Figure 2:**
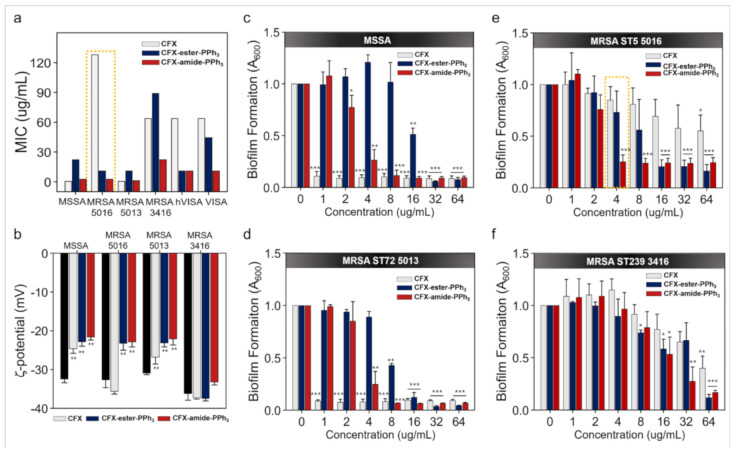
Minimum inhibitory concentration (MIC) and biofilm formation assay for CFX, CFX-ester-PPh_3_, and CFX-amide-PPh_3_ against methicillin-susceptible *S. aureus* (MSSA) and MDR *Staphylococcus aureus* strains. (**a**) MIC values for different compounds with MSSA and five types of MDR *S. aureus*. CFX, CFX-ester-PPh_3_ and CFX-amide-PPh_3_ were serially diluted two-fold in a 96-well microplate according to the concentration range: CFX at 512–0.00005 µg/mL; CFX-ester-PPh_3_ and CFX-amide-PPh_3_ at 178.5–1.39 µg/mL. (**b**) Zeta potential of MSSA and methicillin-resistant *S. aureus* (MRSA) strains. Zeta-potential was measured at 25 °C and determined using phase analysis of scattered light by colloidal particles suspended in de-ionized water (DI H_2_O). (**c**–**f**) Inhibitory activity against biofilm formation depending on concentration of CFX, CFX-ester-PPh_3_, and CFX-amide-PPh_3_. Biofilm formed by MSSA and MRSA strains was stained with crystal violet for 10 min and eluted in 33% acetic acid. The biofilm mass was measured at 600 nm of optical density (OD) values. The yellow triangle indicates maximum inhibition of biofilm formation with CFX-amide-PPh_3_ against the MRSA strains. All compound treated bacteria were statistically calculated compared to the compound untreated group (as a control). The data for the inhibition of biofilm formation in Gram-negative bacteria are represented in Supporting Information (Figure S6). All experiments were repeated three times. The results are shown as the means ± standard deviation of triplicate independent experiments (* *p* < 0.05, ** *p* < 0.001, *** *p* < 0.0001).

As shown in Figure 2a and Table 2, CFX-ester-PPh_3_ and CFX-amide-PPh_3_ showed significant inhibition of bacterial growth at a low concentration range (1.39–89.02 µg/mL) for Gram-positive *S. aureus* and MRSA, both TPP-conjugated CFXs showed better antibacterial activity against sensitive and resistant strains of Gram-positive bacteria than for Gram-negative bacteria. CFX-amide-PPh_3_, in particular, showed excellent antibacterial activity against two strains of MRSA 5016 and 5013 (MIC value = 2.78 and 1.39 µg/mL, respectively). For the MRSA 3416 strain, slightly lower antibacterial activity was observed with MIC values for CFX-ester-PPh_3_ and CFX-amide-PPh_3_ being 89.02 and 22.25 µg/mL, respectively. Interestingly, the MRSA ST5 5016 strain showed resistance towards CFX with an MIC value of 128 µg/mL. However, it showed high sensitivity towards CFX-amide-PPh_3_ and CFX-ester-PPh_3_ with a relatively low MIC value of 2.78 µg/mL and 11.12 µg/mL (Figure 2a and Table 2), respectively. Similarly, vancomycin intermediate-resistant *S. aureus* (VISA) and heterogeneous VISA (hVISA) had high MIC values with CFX (64.0 µg/mL); however, hVISA showed a slightly lower MIC value of 11.12 µg/mL when treated with CFX-ester-PPh_3_ and CFX-amide-PPh_3_. For VISA, treatment with CFX-amide-PPh_3_ showed a lower value of 11.12 µg/mL; however, after treatment with CFX-ester-PPh_3,_ it showed a higher value of 44.51 µg/mL. From these data, we confirmed that TPP-modified CFXs showed a higher antibacterial activity against MRSA; moreover, CFX-amide-PPh_3_ performed better than CFX-ester-PPh_3_ and CFX. As shown in Table 2, CFX derivatives (especially CFX-amide-PPh_3_) showed excellent antibacterial activity against MDR strains compared to original CFX. However, it showed slightly reduced antibacterial activity (high MIC values) against drug-sensitive strains, and this is probably due to the chemical modification of carboxylic acid within CFX and its mode of action changes. From these results, we understand that our chemical conjugation approach on the carboxylic acid within CFX might be promising against MDR bacteria rather than drug-sensitive bacteria. To confirm the synergetic effect of TPP moiety and CFX in a single molecule, we conducted an additional experiment within the MRSA 5016 strain: antibiotic property analysis of (i) heptyltriphenylphosphonium bromide (TPP) only, (ii) scramble of TPP and CFX, (iii) CFX only, (iv) CFX-ester-PPh_3_, and (v) CFX-amide-PPh_3_ (Figure S2a). In this experiment, the set of “TPP only” showed no antibiotic effect against MRSA 5016 strain. No significant antibiotic effect was also observed in the sets of CFX only and the scramble of TPP with CFX (above 65 μg/mL). In the case of TPP-conjugated CFX derivatives, lower MIC values of 11.12 μg/mL and 2.56 μg/mL were observed for the CFX-ester-PPh_3_ and CFX-amide-PPh_3_, respectively. These results represent that the hybrid of TPP moiety and CFX in a single molecule gave a synergetic antibiotic effect in terms of large hydrophobic surface area and delocalized charge distribution of TPP, alter the proton motive force, and regulation of the CFX-related gene expression.

For strains of Gram-negative MDR bacteria, such as carbapenem-resistant *E. coli* (CRE NDM-1 type), carbapenem-resistant *K. pneumoniae* (CRE KPC type), carbapenem-resistant *A. baumannii* (CRAB), and carbapenem-resistant *P. aeruginosa* (CRPA), however, both of the modified CFXs showed no significant antibacterial activity at a concentration ≥178.05 µg/mL (Table 2 and Figure S2b). These data suggest that CFX, modified with TPP moiety, possesses excellent antibacterial activity against Gram-positive bacteria, especially MSSA and MRSA strains. The selectivity for Gram-positive bacteria is expected because of the presence of the bacterial outer membrane (OM), which exists only in Gram-negative bacteria. The OM in Gram-negative bacteria is impermeable to toxic molecules, such as antimicrobial compounds, depending on their molecular weight (MW), and shows limited diffusion of hydrophobic substances via lipopolysaccharide (LPS) on the OM [23,24]. O’Shea and Moser have reported that the cell envelope of Gram-negative bacteria does not allow molecules larger 600 Da to pass through. Large antibiotics, such as vancomycin and daptomycin (~ MW of 1,400 Da), cannot penetrate the OM of Gram-negative bacteria. Therefore, a large hydrophobic surface with high lipophilicity and delocalized charge distribution, such as TPP, and high MW CFX-derivatives (~700 Da) affect the permeability of TPP-conjugated CFXs through the OM layers, these factors could explain the ineffective activity against Gram-negative bacteria [25,26].

### 2.3. Cytotoxicity Assay

In our previous work, the low toxicity of CFX derivatives was verified within various cell lines, including human fibroblast cell line (BJ) and non-tumorigenic breast epithelial cell line (MCF 10A) [21]. According to the report, the CFX-ester-PPh_3_ showed the half maximal inhibitory concentration (IC_50_)value as 631.82 μM toward BJ cell, and >1000 μM for MCF 10A cell, respectively, and these results represent the negligible toxicity of CFX derivatives against normal cells. We additionally performed the hemolysis assay against mouse red blood cells (RBCs) to confirm the effect of the CFX and CFX derivatives toward the hemolysis of MSSA (Figure S3). The non-treated control group (only bacteria) showed the hemolysis of RBCs, approximately 50%, compared with the positive control group (treatment 0.1% of Triton-X 100). In contrast, the compounds-treated groups showed the significantly inhibited hemolysis of RBCs at 2× MIC to less than 2%. In the case of the CFX, the dose-dependent hemolysis of RBCs in MSSA was observed when compared with the non-treated control group. From the results, we confirmed that the CFX-derivatives significantly affect the RBCs hemolysis activity of MSSA.

### 2.4. Membrane-Potential Analysis

As the CFX-derivatives are conjugated with the lipophilic, cationic PPh_3_ moiety, they can rapidly access the negatively charged phospholipid bilayer of the bacterial membrane. We assumed that the membrane-potential of bacterial strains could be increased by the PPh_3_ moiety. To evaluate change in electric charge on the bacterial surface, we measured the zeta-potential of MSSA and MRSA strains. Figure 2b shows the zeta-potential of MSSA and MRSA strains treated with or without CFX and the two CFX-derivatives. The average zeta-potential value of MSSA (no treatment control) was found to be −32.4 mV ± 0.95, indicating a negative membrane charge. In the case of MSSA, treatment with CFX, CFX-ester-PPh_3_, and CFX-amide-PPh_3_ significantly increased zeta-potential values to −24.6 mV ± 1.32, −22.8 mV ± 1.93, and −21.7 mV ± 1.18, respectively. The zeta-potentials for MRSA strains (5016, 5013, and 3416, no treatment control) were found to be −32.7 mV ± 1.75, −30.9 mV ± 0.35, and −36.2 mV ± 1.60, respectively. The zeta-potential for MRSA 5016 strain, treated with CFX alone, was found to be slightly decreased at −35.6 mV ± 0.88 compared to that for the untreated control group; however, in CFX-ester-PPh_3_ and CFX-amide-PPh_3_ treatment groups, MRSA 5016 showed a significantly increased zeta-potential at −23.2 mV ±1.80 and −22.1 mV ±1.60, respectively. The zeta-potential of MRSA 5013 increased significantly to −26.6 mV ± 1.88, −23.1 mV ± 0.97, and −22.1 mV ± 1.60, respectively, for CFX, CFX-ester-PPh_3_, and CFX-amide-PPh_3_ treatment groups. For MRSA 3416 strain, the zeta-potential increased slightly to −33.2 mV ± 0.68 only in the CFX-amide-PPh_3_ treatment group. We monitored the membrane activity mechanism of actions of CFX derivatives by fluorescence spectroscopy analysis. We used a cationic membrane potential-sensitive fluorescence probe, 3,3′-Dipropylthia-dicarbocyanine iodide (DiSC_3_) [27], to monitor the depolarization of the bacteria (MSSA, MRSA) membrane. The fluorescence of DiSC_3_ is increased when it is released into the medium due to the bacteria membrane disruption. We observed that the fluorescence intensity of DiSC_3_ was dramatically increased after treatment of CFX derivatives at the 2.0× and 4.0× MIC (Figure S4), while the CFX-treated group had no increment of fluorescence at any concentration. Thus, the cationic character of PPh_3_ modified CFX derivatives might have more interactions with the negatively charged bacterial membrane, shifting the membrane potential towards neutral. This could contribute to the destabilization of the bacterial membrane [28].

### 2.5. Crystal Violet Assay

We examined the antibiofilm activities of CFX and CFX-PPh_3_ derivatives against 16 species of Gram-negative and Gram-positive bacteria. Similar to the MIC results, CFX-ester-PPh_3_ and CFX-amide-PPh_3_ significantly inhibited biofilm formation in Gram-positive bacteria compared to that in Gram-negative bacteria (Figure 2c–f and Table 2). As shown in Figure 2c–f, biofilm formation of CFX-treated MSSA was inhibited by more than 90% at all concentrations (0.5–256 µg/mL). Compared to CFX alone, CFX-PPh_3_ derivatives showed a slightly lower inhibitory activity against biofilm formation, 48.9% and 73.6% at 16 µg/mL and 4 µg/mL, respectively. Further, CFX showed inhibitory activity against biofilm formation, 54.1% and 60.2% at higher doses of 128 µg/mL and 64 µg/mL, respectively, for MRSA 5016 and 3416 strains. For MRSA 5013 strain, 89.5% inhibitory activity of CFX at 0.5 µg/mL was seen. However, the CFX-amide-PPh_3_ showed excellent inhibitory activity against biofilm formation compared to CFX. The biofilm formation by MRSA 5016 was inhibited to 73.6% by 4 µg/mL CFX-amide-PPh_3_ and to 93.3% by 8 µg/mL CFX-amide-PPh_3_. For MRSA 5013 strain, CFX and CFX-PPh_3_ derivatives showed antibiofilm activity, 88.1% at 0.5 µg/mL (CFX), 57.2% at 8 µg/mL (CFX-ester-PPh_3_), and 75.2% at 4 µg/mL (CFX-amide-PPh_3_) (Figure 2c–f and Table 2). Next, we evaluated the compound’s inhibition property of biofilm formation at the sub-MICs (listed in Table 3) against MSSA and MRSA 5016. The results show that the CFX and CFX-PPh_3_ derivatives effectively inhibited the biofilm formation at 1/2 sub-MIC of MSSA by more than 80%. The biofilm formation in MRSA was inhibited by sub-MIC of CFX or CFX-amide-PPh_3_ more than 40%. The CFX-ester-PPh_3_ slightly inhibited biofilm formation at 1/2 sub-MIC of MRSA to 26.7% (Figure S5a, Table S2). Additionally, we conducted the biofilm assay to confirm whether the CFX derivatives have any ability to disrupt the preformed bacterial biofilm or not. The results show that the CFX derivatives could disrupt the biofilm of MSSA and MRSA, approximately 20%, at higher concentrations of 32 μg/mL and 64 μg/mL, which are slightly higher concentrations than MIC (Figure S5b). Given these results, we verified the properties of CFX derivatives for inhibition of bacterial biofilm formation at sub-MIC and the preformed biofilm’s disruption ability. In the case of the Gram-negative susceptible and resistant bacteria, both the TPP-modified CFXs showed no antibiofilm activity, these results correlate with the MIC results (Figure S6).

### 2.6. Time-Kill Assay

**Figure 3:**
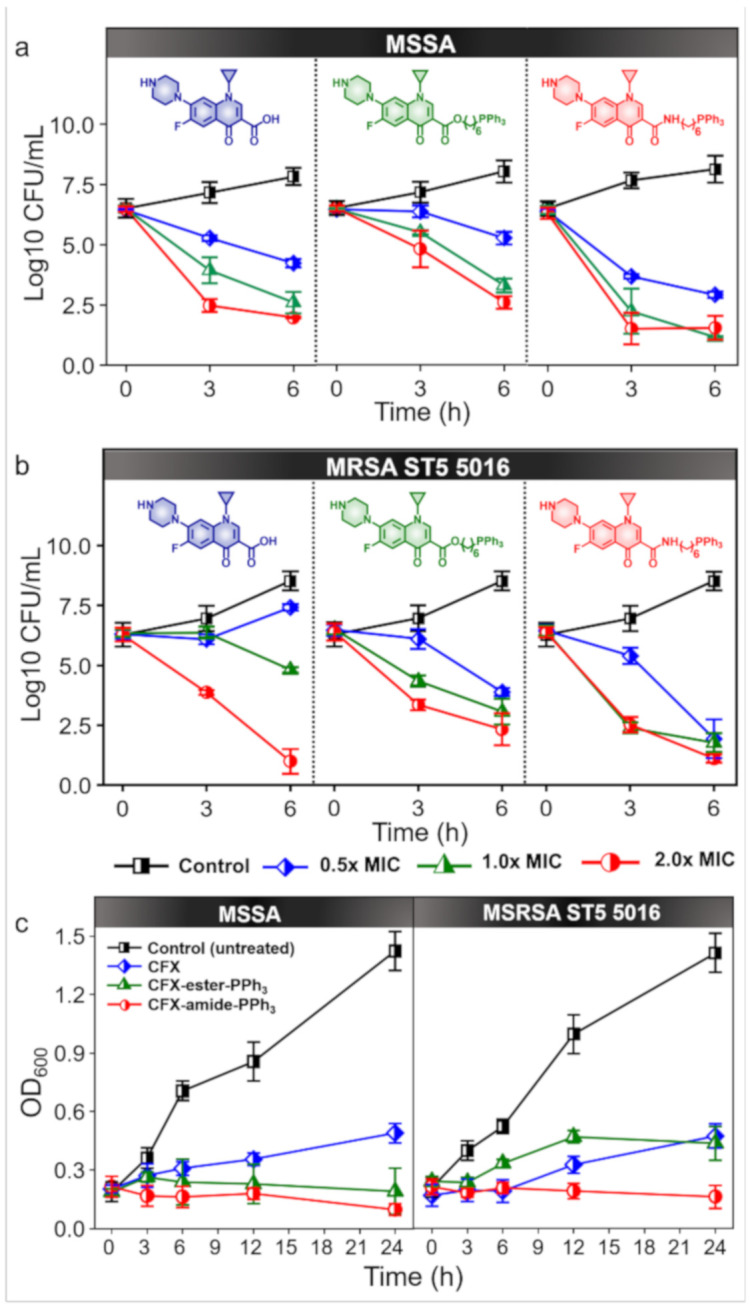
Time-and concentration-dependent efficacy assay for MRSA and MSSA with CFX and CFX-PPh_3_ derivatives. (**a**,**b**) Time-kill assay of CFX and CFX-PPh_3_ derivatives against (**a**) MSSA and (**b**) MRSA. Inset A–C representative concentrations; A: 0.5× MIC, B: 1.0× MIC, C: 2.0× MIC. These data are represented as the means ± standard deviation of three results. (**c**) The growth curve of MSSA and MRSA treated with CFX and CFX-PPh_3_ derivatives at 1.0× MIC of each compound. Each point represents the OD values at 600 nm. All experiments were performed in three independent replicates. The 0.5× MIC, 1.0× MIC, and 2.0× MIC values in this experiment are indicated in Table 2.

Of the two derivatives, CFX-amide-PPh_3_ exhibited superior antibacterial activity against MDR *S. aureus,* such as the MRSA and VISA, with MIC values ranging from 1.36 µg/mL to 22.25 µg/mL. In this assay, we chose the MRSA ST5 5016 strain, which is resistant towards CFX (Figure 3 and Figure S7). MSSA was used as a control. First, a time-kill assay was performed to assess the antibacterial activity of CFX and CFX-PPh_3_ derivatives against MSSA and MRSA ST5 5016 at different concentrations (0.5× MIC, 1× MIC, and 2× MIC, see Methods section for the details and Table 3 for the MIC values).

After the initial inoculation of approximately 1 × 10^6^ colony forming units (CFUs)/mL of MSSA and MRSA, CFX-PPh_3_ derivatives showed a dramatic increase in antibacterial activity within 6 h (Figure 3a,b). CFX-treatment was ineffective against MRSA in the concentration range of 0.5× MIC and 1× MIC within 6 h; however, the CFX-PPh_3_ derivatives showed significant activity at all concentration ranges over the given time. In particular, CFX-amide-PPh_3_ killed approximately 99.9% of MRSA at 2× MIC within 3 h and showed more than 99.9% activity at lower concentration ranges of 0.5× and 1× MIC, within 6 h. In the case of MSSA, CFX and CFX-ester-PPh_3_ treatment at 0.5×, 1×, and 2× MIC significantly decreased the viable cell count within 6 h. Interestingly, CFX-amide-PPh_3_ treatment eliminated MSSA at 2× MIC within 3 h. In terms of MSSA-killing rate, CFX-amide-PPh_3_ was faster than CFX-ester-PPh_3_. These results indicate that the TPP-modified CFX derivatives had high bactericidal activity (eradicating more than 99.9%) against MSSA and MRSA at a concentration of 2× MIC. The inhibitory effects of CFX-PPh_3_ derivatives on the growth of MSSA and MRSA were also confirmed by growth curve measurements (Figure 3c). According to the results, CFX and CFX-PPh_3_ derivatives at 1× MIC effectively inhibited the growth of MSSA and MRSA (decreasing in CFUs/mL value by approximately 100-fold).

### 2.7. Morphology Analysis

**Figure 4:**
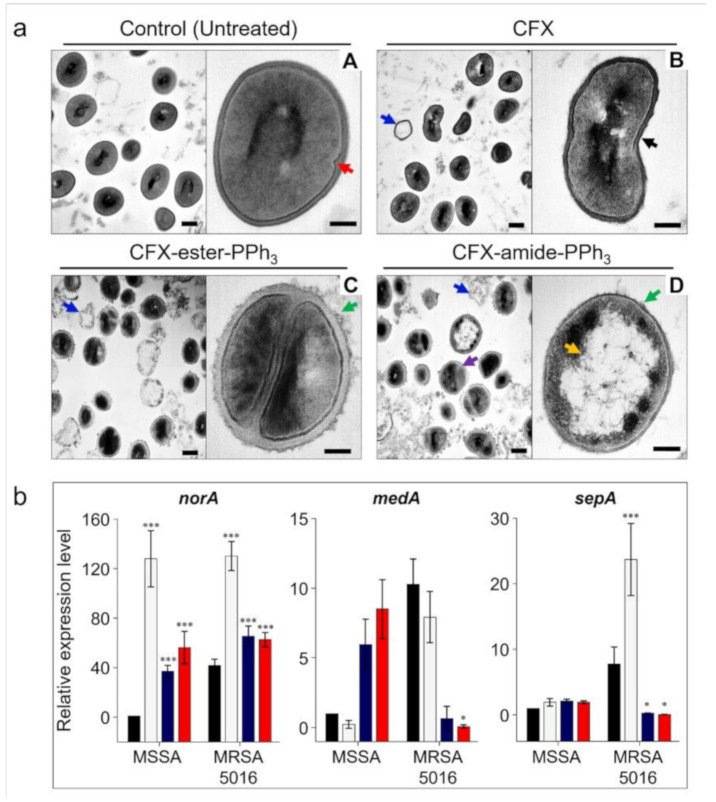
(**a**) Transmission electron microscopy (TEM) images of MRSA ST5 5016 cells treated with each compound at 0.5× MIC for 6 h at different magnifications. A: untreated control of MRSA ST5 5016, B: MRSA treated with 0.5× MIC of CFX, C: MRSA treated with 0.5× MIC of CFX-ester-PPh_3_, and D: MRSA treated with 0.5× MIC of CFX-amide-PPh_3_. The images on the left of all micrographs indicate low-magnification TEM images (low: 40,000× and scale bar: 200 nm) and on the right, high-magnification images of the indicated area (high: 100,000× and scale bar: 100 nm). Each arrow represents the following; the red arrow: intact septum, the black arrow: thinned and irregular cell wall and distortion of the cell, the green arrow: a thorn-like layer present around the outer wall, the purple arrow: partial disintegration of the cytoplasmic membrane, the yellow arrow: loss of cell contents, and the blue arrow: lysis of intracellular contents. (**b**) Effects of CFX and CFX-PPh_3_ derivatives on the relative mRNA expression level of efflux pump genes, such as *norA*, *sepA*, and *mdeA,* in MSSA and MRSA. The gene expression level was measured after CFX and CFX-PPh_3_ derivative treatment at 0.5× MIC in comparison to the drug-free growth used as control. Each cycle threshold (*Ct*) value was normalized to 16S rRNA as internal control, and the normalized fold change was calculated using the delta-delta *Ct* method, with a drug-free group being the control. Gray bar (MSSA) was assigned a value of 1 and represented the control. The results are shown as the means ± standard deviation of triplicate independent experiments. * Significant, *p* < 0.05, *** highly significant, *p* < 0.0001.

From the antibacterial activity analysis and time-kill assay results, the improved properties of TPP-conjugated CFXs were verified for MDR bacteria treatment. The lipophilic and cationic property of TPP enables it to penetrate across the negatively charged membranes of MDR bacteria. To understand the mode of action, such as direct contact with the bacterial cell membrane, we first observed the morphological changes in MRSA using TEM imaging after treating the bacteria with CFX and CFX-PPh_3_ derivatives (Figure 4a). The control set of MRSA (without antibiotics) exhibited a well-defined morphology of coccus with an intact septum and smooth surface features of bacteria (red arrow in Figure 4a, A). However, the antibiotic-treated sets (CFX, CFX-PPh_3_ derivatives) showed significant membrane deformation, with irregular and rough surfaces (Figure 4a, B–D). CFX and CFX-derivatives treated MRSA had a thinner cell wall and the cell shape was distorted (black arrow). A thorn-like layer was present around the outer wall (green arrow). Moreover, a portion of the cytoplasmic membrane of MRSA was in lysis (purple arrow). These findings indicate that both the cell wall and the cytoplasmic membrane of MRSA were affected by CFX-PPh_3_ derivatives, resulting in the loss of cellular contents (yellow arrow) and lysis of intracellular contents (blue arrow). Notably, MRSA treated with CFX-amide-PPh_3_ appeared to have a significantly damaged membrane (Figure 4a, D). We also observed some leakage of the cytoplasmic contents of bacteria to the extracellular environment, due to membrane lysis. From the TEM imaging analysis, we confirmed the working mechanism of TPP-conjugated CFXs, direct contact with the MRSA membrane and disruption of the intact morphology. According to a report by Jan Trnka et al., the lipophilic TPP moiety is capable of accumulating in a negatively charged compartment [29]. Bacterial membranes are composed of highly negatively charged phospholipids, including cardiolipin (CL) and phosphatidyl-glycerol (PG) [30]. Therefore, lipophilic and cationic TPP-modified CFXs selectively target and collapse the anionic bacterial membranes of MSSA and MRSA.

### 2.8. Gene Expression Analysis

To understand the changes in the expression of membrane-related genes, we performed qRT-PCR analysis for the multi-efflux drug pump (MEDP)-related genes. As MEDPs use the proton motive force (PMF) to release the antibiotics, it is possible to prevent the action of MEDPs by decreasing PMF. In this study, we examined the levels of *norA*, *sepA*, and *medA*, which are related to PMF-dependent MEDPs (Figure 4b). We observed a significant over-expression of *norA* mRNA in both MSSA (127.8-fold) and MRSA (130-fold), after CFX treatment, and compared these levels to that for the untreated control. However, the mRNA expression levels of the *norA* were down-regulated in MSSA (CFX-ester-PPh_3_: 37-fold, CFX-amide-PPh_3_: 56.2-fold) and MRSA (CFX-ester-PPh_3_: 65.4-fold, CFX-amide-PPh_3_: 62.6-fold) treated with TPP-modified CFXs compared to the strains treated with CFX. In the case of *sepA* and *medA*, MSSA showed no significant changes for both CFX-PPh_3_ derivatives, but MRSA showed a significantly decreased level of gene expression, *sepA* (CFX-ester-PPh_3_: 0.3-fold, CFX-amide-PPh_3_: 0.1-fold), *medA* (CFX-ester-PPh_3_: 0.7-fold, CFX-amide-PPh_3_: 0.1-fold), compared to the untreated control. As expected, CFX-treated MRSA had increased expression levels of *sepA* (23.7-fold) and *medA* (7.9-fold). A similar increase in the expression level of *norA* (encodes the MDR efflux pump) was observed in MRSA. As MRSA 5016 strain is resistant towards CFX, we observed an over-expression of CFX-related efflux pump genes (*norA*, *sepA*, and *medA*). However, CFX-PPh_3_ derivatives showed down-regulated levels of these genes in wild-type *S. aureus* and MDR *S. aureus*. According to Chang et al. (2018), TPP^+^-conjugated natural products show antifungal activities against *Candida* spp., because TPP^+^ can bypass the drug efflux, as it is not affected by efflux pumps [31]. Based on the results, TPP moiety, due to its inhibition of drug efflux, can help to enhance antibacterial activities of CFX. Additionally, we performed the qPCR analysis of the DNA-gyrase related gene, *gyrA*, to confirm the DNA-gyrase inhibition ability (CFX’s mode of action) of CFX derivatives [18]. We checked the mRNA expression level of the *gyrA* gene in MSSA by qRT-PCR, and the result shows that the expression of the *gyrA* gene was significantly reduced in the CFX derivatives-treated group. Given this result, it can be expected that the CFX derivatives have bacterial DNA-gyrase inhibition ability (<1-fold) similar to CFX (Figure S8).

In the reports, the tolerance acquisition towards CFX in *S. aureus* has shown that the MIC increases four-fold after four passages and eight-fold after eight passages [32]. Unlike these results, the CFX derivatives in our study showed no change in the MIC values until eight passages in the same experimental conditions. The CFX resistance of *S. aureus* has genetically evolved via the acquisition of mutations in the *gyrA* or the *norA* gene through the following: (i) accelerating the multidrug efflux pumps (MDEPs) such as *norA*, *sepA*, and *medA,* (ii) generating mutations at the quinolone resistance-determining regions (QRDRs) to reduce the affinity of the CFX [33]. We concluded that the CFX-PPh_3_ derivatives have excellent antibacterial activity by inhibition of DNA gyrase and efflux pump ability. These results suggest that the CFX-PPh_3_ derivatives could affect the delay in the acquisition of resistance more than CFX. TPP-conjugated CFX derivatives were prepared using previously published protocols (details in Supporting information) [21].

## 3. Materials and Methods

### 3.1. Bacteria Strains and Culture

All strain-related studies were conducted in certified biosafety level (BSL) facilities at the Kyung Hee University Medical Center (Seoul, Republic of Korea). All strain-related information has been listed in Table 1. Strains of drug-sensitive bacteria (control strain) were obtained from American Type Culture Collection (ATCC, Manassas, VA, USA) and Culture Collection of Antibiotic Resistant Microbes (CCARM, Seoul, Republic of Korea). Strains of drug-resistant bacteria were obtained from Asan Medical Center (ASM, Seoul, Republic of Korea). All bacterial strains were stored in skimmed milk and frozen at −70 °C. The bacterial strains were sub-cultured twice in cation-adjusted Mueller–Hinton broth (CA-MHB) for 24 h at 37 °C, prior to minimum inhibitory concentration (MIC) analysis and time or concentration-dependent study.

### 3.2. Preparation of CFX-PPh_3_ Derivatives

TPP-conjugated CFX derivatives were prepared using previously published protocols (details in Supporting Information) [21].

### 3.3. MIC Assay

MIC was determined by using broth microdilution in CA-MHB, according to the Clinical and Laboratory Standard Institute (CLSI, 2016) guidelines [23]. In this study, we performed the MIC assay for 16 types of strains, including MDR bacteria against CFX, CFX-ester-PPh_3_, and CFX-amide-PPh_3_. Briefly, CFX and CFX-PPh_3_ derivatives were serially diluted (two-fold) using CA-MHB broth in a 96-well microplate. The turbidity of all strains was adjusted to a 0.5 McFarland standard (1 × 10^8^ CFU/mL), 10 μL of bacterial suspension was added to each well of a 96-well microplate, and the final concentration of each strain was approximately 5 × 10^5^ CFU/mL. The contents of the microplate were mixed well and incubated at 37 °C for 20 h. Thereafter, the lowest concentration of CFX and CFX-PPh_3_ derivatives, with no growth, was taken as the MIC value. For the MIC assay, the typpe strain obtained from the microorganism bank was used as the quality control strain. Each experiment was conducted in triplicate.

### 3.4. Membrane-Potential Analysis

The membrane-potential of methicillin-sensitive *S. aureus* (MSSA) and MRSA strains was measured by zeta-potential analysis, as reported previously by Halder et al. [34]. Briefly, all strains were grown in CA-MHB at 37 °C overnight, 0.1% of culture media were inoculated into 3 mL of fresh CA-MHB, and the bacteria were cultured at 37 °C until the density reached approximately 1 × 10^7^ CFU/mL. Thereafter, 4× MIC (Table 3) of CFX and CFX-derivatives were added to the culture media, which were incubated in a shaking incubator at 200 rpm for 6 h at 37 °C. Subsequently, 1 mL of bacterial suspension was centrifuged at 13,000 rpm for 5 min, and the supernatant was removed. The pellets were washed three times with de-ionized water (DI H_2_O) and re-suspended in 1 mL of DI H_2_O. Finally, the bacterial suspension was diluted 10-fold in DI H_2_O immediately prior to zeta-potential measurement. The zeta-potential of bacterial membranes was measured using Zetasizer Nano ZS90 (Malvern Instruments, Malvern, UK), equipped with a helium-neon laser (633 nm) as a light source, at 25 °C. Each measurement was repeated three times, and all experiments were performed in two technical replicates.

### 3.5. Biofilm Formation Assay

Inhibition of biofilm formation, of 16 species of ESKAPE bacteria including MDR strains, was investigated using crystal violet staining [35]. First, all 16 types of bacterial strains were cultured in tryptic soy broth (TSB; BD Difco, product no. 211825, Franklin Lakes, NJ, USA) at 37 °C overnight, the culture was resuspended in fresh TSB to obtain 0.5 McFarland turbidity. After 10-fold dilution in TSB, 200 μL TSB with 0.1% glucose and 10 μL bacterial suspension (approximately 5 × 10^5^ CFU/well) was seeded into individual wells in a flat-bottomed 96-well polystyrene microwell plate (Corning Costar, product no. 3365, Glendale, ARI. USA). To screen for anti-biofilm activity, CFX and CFX-PPh_3_ derivatives were added to the bacterial suspension in concentrations ranging from 0.5–512 μg/mL, and the plates were incubated at 37 °C for 24 h. Thereafter, the culture broth and planktonic cells were removed carefully, and the wells were rinsed with phosphate-buffered saline (PBS, pH 7.4) three times and completely dried at 50 °C for 2 h. The dried plates were stained with 1.0% crystal violet for 10 min at room temperature and gently rinsed with DI H_2_O. At this point, the biofilm biomass was observed as a purple ring on the wall of each well. For quantification of the biofilm biomass, 200 μL of 33% glacial acetic acid was added to each well and incubated for 20 min with shaking. The optical densities of the stained biofilm were measured at 600 nm using a microplate reader (Spark 10M, Tecan, Crailshim, Germany). Average absorbance for each bacterial strain was determined, and percentage inhibition of biofilm formation was calculated using Equation (1). Biofilm formation assay was performed in three biological replicates, each consisting of two technical replicates. Equation (1):

Inhibition (%) = OD_Positive-control_ – OD_Experimental_/OD_Positive-control_. (1)

### 3.6. Time-Kill Assay

All the strains were cultured overnight on blood agar plates, as per the standard protocol. Thereafter, the turbidity of all strains was adjusted to 0.5 McFarland standard, and the strains were inoculated at 0.1% in CA-MHB and cultured at 37 °C for 3 h with shaking (150 rpm). CFX and CFX-PPh_3_ derivatives were added to the cultures when a density of approximately 10^6^ CFU/mL was reached. The concentrations of compounds used for the time-kill assay were based on MIC results (Table 3): (i) MSSA: (CFX) 0.5× (0.25 µg/mL), 1× (0.5 µg/mL), and 2× (1.0 µg/mL); (CFX-ester-PPh_3_) 0.5× (11.12 µg/mL), 1× (22.25 µg/mL), and 2× (44.51 µg/mL); (CFX-amide-PPh_3_) 0.5× (1.39 µg/mL), 1× (2.78 µg/mL), and 2× (5.56 µg/mL). (ii) MRSA: (CFX) 0.5× (64 µg/mL), 1× (128 µg/mL), and 2× (256 µg/mL); (CFX-ester-PPh_3_) 0.5× (5.56 µg/mL), 1× (11.12 µg/mL), and 2× (22.25 µg/mL); (CFX-amide-PPh_3_) 0.5× (1.39 µg/mL), 1× (2.78 µg/mL), and 2× (5.56 µg/mL). To record measurement of kill (log_10_ scale of viable cells), samples (10 µL) that were taken at each time point (0, 3, and 6 h) were serially diluted with sterile DI H_2_O and spread on drug-free Luria-Bertani (LB) plates. After a 24 h incubation period at 37 °C, the colonies were counted.

### 3.7. Quantitative Real-Time PCR Analysis

mRNA expression of CFX resistance-related genes (*norA*, *sepA*, and *mecA*) was measured using qRT-PCR. To obtain total RNA, MSSA and MRSA were cultured in CA-MHB with or without CFX and CFX-PPh_3_ derivatives (0.5× MIC) at 37 °C for 24 h with shaking (150 rpm). RNA was extracted using the easy-BLUE^TM^ Total RNA Extraction kit (iNtRON Biotechnology, Republic of Korea), according to the manufacturer’s instructions. Thereafter, cDNA was synthesized using the High-Capacity cDNA Reverse Transcription kit (Applied Biosystems, product no. 43-688-14, Foster, CA, USA). Applied Biosystems 7300 Real-Time PCR (Applied Biosystems, Foster, CA, USA) was used for gene expression analysis of the target genes with 2× KAPA Master mix SYBR^®^ (Kapa Biosystems, product no. KR0389, San Francisco, CA, USA). qPCR cycling was performed at 95 °C for 10 min, followed by 40 cycles at 95 °C for 3 s, and finally at 60 °C for 30 s. Gene-specific amplification was confirmed by the melting curve. The primer sequences used in this study have been listed in Table S1 (Supplementary Material). Data were normalized using the internal control gene, 16S rRNA, and relative mRNA expression of genes (*norA*, *sepA*, and *mdeA*) was calculated using the 2^−ΔΔCt^ method [36]. Relative gene expression analysis was repeated three times in different biological and technical experiments.

### 3.8. Transmission Electron Microscopy (TEM) Imaging

The morphological changes in MRSA ST5 5016, after treatment with CFX and CFXs-PPh_3_ derivatives, were evaluated using TEM, using a previously described method [37]. MRSA 5016 was grown in CA-MHB overnight until reaching the mid-exponential phase, diluted in fresh CA-MHB at a ratio of 1:10, and cultured for 3 h at 37 °C. Thereafter, MRSA (in CA-MHB) was treated with 0.5× MIC of CFX, CFX-ester-PPh_3_, and CFX-amide-PPh_3_ (Table 3) for 6 h. Subsequently, 5 mL of culture was centrifuged at 13,000 rpm for 5 min, the supernatant was removed, and the cell pellets were thoroughly washed 3 times with 1× PBS. The pellets were then fixed in Karnovsky’s fixative [38] at 4 °C overnight. The pellets were washed thrice with 0.05 M sodium cacodylate buffer at 25 °C, post-fixed in 1% osmium tetroxide (OsO_4_) in 0.1 M sodium cacodylate buffer at 4 °C for 2 h, and washed twice in sterile-distilled water at 25 °C. The washed cell pellets were stained en bloc with 0.5% uranyl acetate at 4 °C overnight and dehydrated using highly pure ethanol (50%, 70%, 80%, 90%, and 100%). Finally, the pellets were treated with 100% propylene oxide for transit and polymerized with propylene oxide and Spurr’s resin in a specific ratio (1:1 and 1:2, respectively). The samples were sectioned using an ultramicrotome equipped with a diamond blade and stained with 3% uranyl acetate on the grid. The stained grid was visualized using the JEM-1010 electron microscope at 80 kV.

## 4. Conclusions

In summary, we have designed TPP-conjugated CFX derivatives, which are chemically conjugated through an ester (CFX-ester-PPh_3_) or an amide bond (CFX-amide-PPh_3_), to alleviate the resistance of CFX, and investigated their antibacterial activity against multi-drug resistant bacteria ESKAPE (16 types of resistant and susceptible strains). The antibacterial activity of CFX-ester-PPh_3_ and CFX-amide-PPh_3_ were systematically analyzed using the MIC assay, TEM analysis, and gene expression analysis by qRT-PCR. The antibacterial activity of CFX-ester-PPh_3_ and CFX-amide-PPh_3_ against Gram-positive bacteria, such as *S. aureus* (MDR *S. aureus* (MRSA isolates)) was excellent. In particular, the MIC values of CFX-ester-PPh_3_ (11.12 µg/mL) and CFX-amide-PPh_3_ (2.78 µg/mL) were significantly lower towards MRSA ST5 5016 strain than CFX alone (128 µg/mL). In the mode of action study, a significant morphological change of MRSA, treated with CFX-amide-PPh_3_, was observed with cell membrane deformation accompanied by irregular and rough surfaces. The mRNA expression level of multi-drug pump-related genes, such as *norA*, *sepA*, and *medA,* in MRSA was analyzed using qRT-PCR, and the level of these genes was observed to dramatically decrease after treatment with the CFX-PPh_3_ derivatives. With these promising data, we concluded that conjugation of the TPP^+^ moiety to traditional antibiotics (CFX in this study) could enhance antibacterial activities against drug-sensitive bacteria, as well as drug-resistant bacteria. Our rationale might be simple but can have a significant impact in the field of medicinal chemistry. This work will be a foundation for the development of new types of antibiotics for the regulation of MDR bacteria. We have an ongoing project based on the piperazine site modified CFX derivatives with various chemical moieties varying in size, charge, and hydrophobicity, and the results will be reported somewhere.

## Figures and Tables

**Table 1:** A list of bacterial strains used in this study. Control: drug-sensitive strains.

Species	Strains	Drug Sensitive or Resistant Bacteria
**Gram-negative**		
*Escherichia coli*	ATCC^®^ 25922	Carbapenem-susceptible *E. coli*
	AMCEC 22365	Carbapenem-resistant *E. coli* (NDM-1 type)
*Klebsiella pneumoniae*	ATCC^®^ 13883	Carbapenem-susceptible *K. pneumoniae*
	AMCKP 24272	Carbapenem-resistant *K. pneumoniae* (KPC type)
*Pseudomonas aeruginosa*	ATCC^®^ 27853	Carbapenem-susceptible *P. aeruginosa* (CSPA)
	CCARM 2321	Carbapenem-resistant *P. aeruginosa* (CRPA)
*Acinetobacter baumannii*	ATCC^®^ 19606	Carbapenem-susceptible *A. baumannii* (CSAB)
	AMCAB 643	Carbapenem-resistant *A. baumannii* (CRAB)
**Gram-positive**		
*Enterococcus faecium*	ATCC^®^ 29212	Vancomycin-susceptible *E. faecium* (VSE)
	CCARM 5024	Vancomycin-resistant *E. faecium* (VRE)
*Staphylococcus aureus*	ATCC^®^ 29213	Methicillin-susceptible *S. aureus* (MSSA)
	AMCSA 5016	Methicillin-resistant *S. aureus* (MRSA, ST5)
	AMCSA 5013	MRSA (ST72)
	AMCSA 3416	MRSA (ST239)
	ATCC^®^ 700698	Heterogeneous VISA (hVISA)
	ATCC^®^ 700699	Vancomycin intermediate-resistant *S. aureus* (VISA)

**Table 2:** Minimum inhibitory concentration values and inhibitory concentration for biofilm formation against *Enterococcus faecium*, *Staphylococcus aureus*, *Klebsiella pneumoniae*, *Acinetobacter baumannii*, *Pseudomonas aeruginosa*, and *Enterobacter* spp. (ESKAPE) pathogens (a total of 16 types), including MDR bacteria, against CFX and CFX-PPh_3_ derivatives. * The biofilm inhibition rate was calculated using the biofilm biomass in bacteria treated with the compounds. Control (untreated strain): biofilm biomass in untreated bacteria. Units: µg/mL. The minimum inhibitory concentration (MIC) values refer to the Clinical and Laboratory Standards Institute, 2016 (CLSI) guidelines.

Type or Strain	CLSI Breakpoint (μg/mL)	QC Range	MIC (μg/mL)			Biofilm Inhibition Rate (%) *		
			CFX	CFX- Ester-PPh_3_	CFX- Amide-PPh_3_	CFX	CFX- Ester-PPh_3_	CFX- Amide-PPh_3_
**Gram-negative Bacteria**								
*E. coli*	≤1	0.004–0.015	0.004	5.56	5.56	<0.5 (96.49%)	32 (76.0%)	8 (85.4%)
NDM-1 type	≤1	-	16	>178.05	89.02	-	-	-
*K. pneumoniae*	≤1	-	0.031	22.25	44.51	-	-	-
KPC type	≤1	-	32	>178.05	178.05	128 (74.8%)	>512 (28.5%)	256 (51.2%)
*P. aeruginosa*	≤1	-	0.25–0.125	89.02	89.02	2 (73.2%)	64 (50.1%)	256 (54.0%)
CRPA	≤1	0.25–2.0	32	>178.05	178.05	<0.5 (80.3%)	64 (74.4%)	32 (56.5%)
*A. baumannii*	≤1		0.5	89.02	89.02	256 (66.0%)	>512 (48.2%)	256 (53.4%)
CRAB	≤1		64	89.02	89.02	-	-	-
**Gram-positive Bacteria**								
*E. faecium*	≤1	0.25–2.0	1.0-0.5	178.05	44.51	<0.5 (65.0%)	64 (60.0%)	64 (91.5%)
VRE	≤1	-	256	89.02	22.25	-	-	-
*S. aureus*	≤1	0.12–0.5	0.5	22.25	2.78	<0.5 (89.49%)	16 (48.9%)	4 (73.6%)
MRSA 5016	≤1		128	11.12	2.78	128 (54.1%)	8 (43.9%)	4 (74.9%)
MRSA 5013	≤1		0.5	11.12	1.39	<0.5 (88.1%)	8 (57.2%)	4 (75.2%)
MRSA 3416	≤1		64	89.02	22.25	64 (60.2%)	64 (88.2%)	32 (72.5%)
hVISA	≤1		64	11.12	11.12	-	-	-
VISA	≤1		64	44.51	11.12	-	-	-

**Table 3:** The MIC values of CFX and CFX-PPh_3_ derivatives. The values were used for the analysis of zeta-potential, time-kill assay, OD measurement, TEM imaging, and qRT-PCR analysis. Units: µg/mL.

MIC	MSSA			MRSA ST5 5016		
	CFX	CFX-ester-PPh_3_	CFX-amide-PPh_3_	CFX	CFX-ester-PPh_3_	CFX-amide-PPh_3_
0.5×	0.25	11.12	1.39	64	5.56	1.39
1.0×	0.5	22.24	2.78	128	11.12	2.78
2.0×	1.0	44.48	5.56	256	22.24	5.56
4.0×	2.0	89.96	11.12	512	44.48	11.12
8.0×	4.0	178	22.25	1,024	88.96	22.24

## Supplementary Materials

The following are available online at https://www.mdpi.com/2079-6382/9/11/758/s1, Figure S1: Minimum inhibitory concentration (MIC) assay using a 96-well plate of CFX and CFX derivatives for 16 types of MDR bacteria, Figure S2: MIC assay against Gram-negative and Gram-positive bacteria strains, Figure S3: The effect of CFX and CFX-PPh_3_ derivatives on hemolysis of MSSA and MRSA, Figure S4: The cytoplasmic membrane depolarization against MSSA and MRSA by CFX and CFX-PPh_3_ derivatives, Figure S5: Biofilm formation assay using crystal violet against 16 types of MDR bacteria, Figure S6: Biofilm formation assay at sub-MIC of CFX and CFX-PPh_3_ derivatives against MSSA and MRSA, Figure S7: LB agar plate showing MSSA and MRSA CFU treated with CFX and CFX derivatives, Figure S8: Effects of CFX and CFX-PPh_3_ derivatives on the expression level of *gyrA* gene in MSSA, Table S1: Primer list, Table S2: Biofilm formation inhibition rate at ½ sub-MIC of CFX and CFX-PPh_3_ derivatives.
