# Supplementary material for: Membrane-Targeting Triphenylphosphonium Functionalized Ciprofloxacin for Methicillin-Resistant Staphylococcus aureus (MRSA)
Source: Antibiotics (Basel). 2020 Oct 30;9(11):758. doi: 10.3390/antibiotics9110758 (PMC7693559; doi:10.3390/antibiotics9110758)
Supplement: Supplementary file 1 [file antibiotics-09-00758-s001.pdf]

Supplementary Information

Membrane-Targeting Triphenylphosphonium Functionalized Ciprofloxacin for Methicillin-Resistant *Staphylococcus aureus* (MRSA)

Sangrim Kang,† Kyoung Sunwoo,† Yuna Jung, Junho K. Hur, Ki-Ho Park,* Jong Seung Kim,* and Dokyoung Kim*

† These authors contributed equally to this work

*Correspondence should be addressed to: dkim@khu.ac.kr (D.K.), jongskim@korea.ac.kr (J.S.K.), kiho@khu.ac.kr (K.P.)

Table of Contents

1. Materials and Methods

- General Information
- Preparation of CFX derivatives
- Hemolysis Assay
- Membrane depolarization assay

2. Supporting Figures and Tables

- Figure S1. Minimum inhibitory concentration (MIC) assay using a 96-well plate of CFX and CFX derivatives for 16 types of MDR bacteria
- Figure S2. MIC assay against Gram-negative and Gram-positive bacteria strains
- Figure S3. The Effect of CFX and CFX-PPh_3 derivatives on hemolysis of MSSA and MRSA
- Figure S4. The cytoplasmic membrane depolarization against MSSA and MRSA by CFX and CFX-PPh_3 derivatives
- Figure S5. Biofilm formation assay at sub-MIC of CFX and CFX-PPh_3 derivatives against MSSA and MRSA
- Firugre S6. Biofilm formation assay using crystal violet against 16 types of MDR bacteria
- Firugre S7. LB agar plate showing MSSA and MRSA CFU treated with CFX and CFX derivatives
- Firugre S8. Effects of CFX and CFX-PPh_3 derivatives on the expression level of *gyrA* gene in MSSA

3. Supporting Tables

- Table S1. Primer list
- Table S2. Biofilm formation Inhibition rate at ½ sub-MIC of CFX and CFX-PPh_3 derivatives

4. Supporting References

1. Materials and Methods

General information

The chemical reagents were purchased from Aldrich (USA) and TCI (Japan). The ciprofloxacin (CFX) was purchased from TCI (Product No. C2510, Japan). TPP (heptyltriphenylphosphonium bromide) was purchased from TCI (Product No. H0545, Japan). Commercially accessible reagents and anhydrous solvents were used without further purification. Chemical reactions were carried out under an argon atmosphere. Thin-layer chromatography (TLC) was performed using pre-coated silica gel (60F-254) glass plate from Merck (Product No. 105554, Germany). The Cation-adjusted Mueller Hinton broth (CA-MHB) for bacteria culture was purchased from BD/BBL (Product No. 743-23106, USA). The plasticwares, such as a 96-well microtiter plate (round bottom, Product No. 34296) and conical tube (15 mL/50 mL, Product No. 50115/50050) were purchased from SPL Life Sciences (Rep. of Korea). For RNA extraction of bacteria, the easy-BLUETM RNA extraction kit was purchased from iNtRON Biotechnology (Product No. 17061, Rep. of Korea), and all reagents for qRT-PCR were purchased from Sigma-Aldrich (USA) and Invitrogen (USA). The ABI Step One real-time PCR system (Applied Biosystems, Product No. 4376357, USA) was used for qRT PCR. Transmission electron microscope (TEM) imaging was carried out using JEM-1010 (operating 80 kV, JEOL, Japan).

Preparation of CFX derivatives

All synthetic steps requiring anhydrous conditions were carried out in oven-dried flasks flushed with argon gas, and commercial anhydrous solvents were used. All chemicals were obtained from commercial suppliers and used as it is (Alfa Aesar, Sigma Aldrich and TCI Chemicals). Thin layer chromatography was performed on glass plates pre-coated with Merck 60 silicagel F_{254} plates. Column chromatography was performed using Merck 60 (particle size 0.040-0.063 mm) silica gel. NMR spectral analyses were performed with a Bruker NMR instrument (500 MHz for ^{1}H, 125 MHz for ^{13}C) using MeOD, $CDCl_3$ or DMSO-d_6 as the solvents, and TMS as an internal standard. Chemical shifts (δ) are recorded in ppm and coupling constants are given in Hz. ESI mass spectral analyses were carried out using an LC/MS-2020 Series instrument (Shimadzu, Japan). DMSO for molecular biology was obtained from Sigma Aldrich (St. Louis, Mo, USA) and was used for spectral and biological studies. The Dowex® 1X8 chloride form ion exchange resin was prepared using the following procedure: 20 mL of dry Dowex® 1X8 chloride form beads was added to 100 mL MeOH and stirred gently for 20 min, after which the solvent was decanted, and the procedure was repeated 3 times.

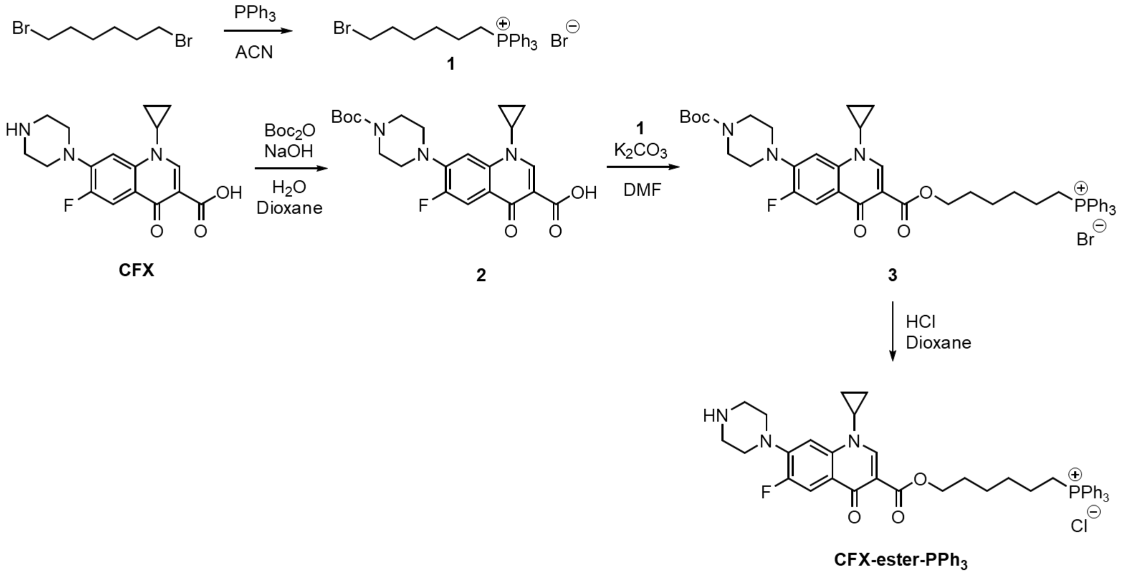

Scheme S1. Synthetic scheme for CFX-ester-PPh_3.

Triphenylphosphonium alkylbromide (1): Compound 1 was synthesized analogously by following the procedure from 1,6-dibromohexane [S1]. Analytical data matched those reported in the literature.

Boc-protected Ciprofloxacin (2): Compound 2 was synthesized analogously by the literature procedure from ciprofloxacin (CFX) [S2]. All analytical data matched those reported in the literature.

Boc-protected CFX-ester-PPh_3 (3): Compounds 1 (1 g, 1.975 mmol) and 2 (852 mg, 1.975 mmol) were dissolved in *N*,*N*-dimethylformamide (DMF, 50 mL), and K_2CO_3 (819 mg, 5.925 mmol) was added to the solution. The reaction mixture was heated overnight to 50 °C. After removing the solvent under reduced pressure, the residue was purified with column chromatography (eluent: DCM (dichloromethane): MeOH (methanol) = 97:3, *v*/*v*). The product was first dissolved in 30 mL MeOH, and 270 mL of distilled water was added. 1 g $NaBF_4$ (9.108 mmol) was added to the solution whilst vigorously stirring for 1 hr. The mixture was then extracted with DCM (3 × 200 mL), the combined organic layers were dried over Na_2SO_4, and the solvent was evaporated under reduced pressure. The resulting BF_4 salt was dissolved in MeOH and 20 mL of Dowex® 1X8 chloride form (prepared as described above), it was then added as the mixture was stirred gently. After the ion exchange finished, the mixture was filtered through a paper filter, and the filtrate was evaporated. 1.31 g of compound 3 (82%) was obtained; ^1H NMR ($CDCl_3$, 500 MHz): δ 8.50 (s, 1H), 7.82–7.77 (m, 3H), 7.74–7.66 (m, 13H), 7.33 (d, *J* = 7.2 Hz, 1H), 4.25 (t, *J* = 6.0 Hz, 2H), 3.65 (t, *J* = 4.8 Hz, 4H), 3.56–3.50 (m, 1H), 3.39–3.29 (m, 2H), 3.22 (t, *J* = 4.9 Hz, 4H), 1.74–1.63 (m, 6H), 1.58–1.51 (m, 2H), 1.50 (s, 9H), 1.37 (dd, *J* = 14.2 and 6.9 Hz, 2H) and 1.11–1.06 (m, 2H) ppm; ^{13}C NMR ($CDCl_3$, 125 MHz): δ 173.0, 165.5, 154.6, 153.2 (d, *J* = 247.7 Hz), 148.2, 144.4 (d, *J* = 10.4 Hz), 138.1, 135.1, 133.6 (d, *J* = 10.0 Hz), 130.5 (d, *J* = 12.7 Hz), 122.9 (d, *J* = 6.6 Hz), 118.1 (d, *J* = 85.4 Hz), 112.7 (d, *J* = 22.9 Hz), 110.0, 105.5, 80.1, 64.3, 53.5, 49.9 (d, *J* = 4.2 Hz), 34.9, 29.5 (d, *J* = 16.0 Hz), 28.4, 28.0, 25.0, 22.3 (d, *J* = 50.3 Hz), 22.1 (d, *J* = 4.3 Hz) and 8.2 ppm; ESI-MS (*m*/*z*): $[M]^+$ calcd for $C_{46}H_{52}FN_3O_5P$: 776.36; found: 776.35.

CFX-ester-PPh_3: Compound 3 (1 g, 1.231 mmol) was dissolved in 30 mL of solvent (1,4-dioxane: DCM, 4:1, *v*/*v*), and 10 mL of anhydrous 4N HCl in dioxane was added dropwise in an ice bath, in order to obtain a final HCl concentration of 1N. The solution was stirred overnight at room temperature, and the solvent was evaporated. CFX-ester-PPh_3 was obtained as a yellowish powder: yield 90% (790 mg, 1.11 mmol). For biological assays, it was further purified with flash column (eluent 10% MeOH in DCM to 12% MeOH in DCM). ^1H NMR ($CDCl_3$, 500 MHz) δ 8.51 (s, 1H), 7.85–7.66 (m, 16H), 7.33 (d, *J* = 7.1 Hz, 1H), 4.27 (t, *J* = 5.8 Hz, 2H), 3.73–3.61 (m, 2H), 3.55–3.50 (m, 1H), 3.43–3.37 (m, 4H), 3.23–3.19 (m, 4H), 1.75–1.67 (m, 6H), 1.61–1.52 (m, 2H), 1.41–1.34 (m, 2H), 1.15–1.08 (m, 2H) ppm.; ^{13}C NMR (DMSO-d6,125 MHz): δ 171.8, 164.8, 152.6 (d, *J* = 246.8 Hz), 148.2, 144.1 (d, *J* = 10.1 Hz), 138.1, 134.9 (d, *J* = 2.7), 133.6 (d, *J* = 10.1 Hz), 130.3 (d, *J* = 12.6 Hz), 121.7 (d, *J* = 6.8 Hz), 118.6 (d, *J* = 86.2 Hz), 111.5 (d, *J* = 22.8 Hz), 109.1, 106.1 (d, *J* = 2.8 Hz), 63.7, 49.7 (d, *J* = 4.1 Hz), 44.7, 34.9, 29.2 (d, *J* = 16.9 Hz), 27.8, 24.5, 21.6 (d, *J* = 4.3 Hz), 20.1 (d, *J* = 49.9 Hz) and 7.6 ppm; ESI-MS (*m*/*z*): $[M]^+$ calcd for $C_{41}H_{44}FN_3O_3P$: 676.31; found: 676.35.

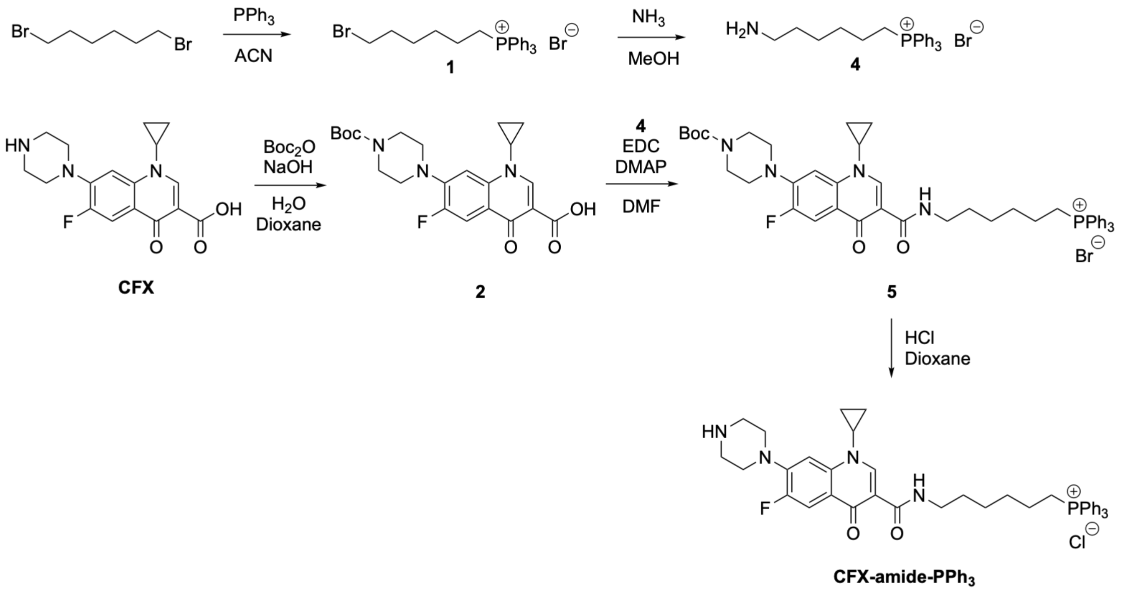

Scheme S2. Synthetic scheme for CFX-amide-PPh3.

Triphenylphosphonium alkylamine (4): Compound 4 was synthesized analogously by following the procedure in the literature [S3]. All analytical data matched those reported in the literature.

Boc-protected CFX-amide-PPh3 (5): Compound 2 (1 g, 2.318 mmol), EDC (1-Ethyl-3-(3-dimethylaminopropyl)-carbodiimide) hydrochloride (667 mg, 3.477 mmol), 1-Hydroxy-benzotriazole hydrate (470 mg, 3.477 mmol) were added to DMF in an ice-bath and stirred for 30 minutes at room temperature. DMAP (4-Dimethylaminopyridine, 425 mg, 3.477mmol) and compound 4 (1.025 g, 2.318 mmol) were added to the flask and stirred overnight. The reaction mixture was then evaporated under reduced pressure. The residue was purified with silica column chromatography. (eluent DCM:MeOH = 97:3, *v*/*v*). The product was first dissolved in 30 mL MeOH, and 270 mL distilled water was added. 1 g $NaBF_4$ (9.108 mmol) was added to the solution whilst vigorously stirring it for 1 hr. The mixture was then extracted with DCM (3 × 200 mL), the combined organic layers were dried over Na_2SO_4, and the solvent was evaporated under reduced pressure. The resulting BF_4 salt was dissolved in MeOH and 20 mL of Dowex® 1X8 chloride form, and was then added to the mixture and stirred gently. After the ion exchange finished, the mixture was filtered through a paper filter, and the filtrate was evaporated. 1.47 g of compound 5 (78%) was obtained. ^1H NMR ($CDCl_3$, 500 MHz): δ 8.77 (s, 1H), 7.97–7.65 (m, 16H), 7.32 (d, *J* = 7.1 Hz, 1H), 3.84–3.74 (m, 2H), 3.66–3.58 (m, 4H), 3.50–3.46 (m, 1H), 3.38–3.31 (m, 2H), 3.24–3.16 (m, 4H), 1.67–1.59 (m, 4H), 1.52–1.48 (m, 2H), 1.46 (s, 9H), 1.38–1.29 (m, 4H) and 1.13–1.09 (m, 2H) ppm.

CFX-amide-PPh3: Compound 5 (1 g, 1.232 mmol) was dissolved in 30 mL of solvent (1,4-dioxane: DCM, 4:1, *v*/*v*), and 10 mL of anhydrous 4N HCl in dioxane was added dropwise in an ice bath, in order to obtain a final HCl concentration of 1N. The solution was then stirred overnight at room temperature, and the solvent was evaporated. CFX-amide-PPh3 was obtained as a yellowish powder: yield 93% (815 mg, 1.15 mmol). For biological assays, it was further purified with flash column (eluent 10% MeOH in DCM to 12% MeOH in DCM). ^1H NMR (MeOD, 500 MHz): δ 8.88 (s, 1H), 7.95–7.74 (m, 17H), 7.69 (d, *J* = 7.2 Hz, 1H) 3.85–3.79 (m, 1H), 3.69–3.64 (m, 4H), 3.54–3.49 (m, 4H), 3.49–3.41 (m, 4H), 1.77–1.70 (m, 2H), 1.70–1.58 (m, 4H), 1.53–1.42 (m, 4H) ppm. ^{13}C NMR (MeOD, 125 MHz): δ 176.5, 167.2, 155.0 (d, *J* = 250.2 Hz), 148.5, 145.6 (d, *J* = 10.7 Hz), 140.3, 136.4 (d, *J* = 3.1 Hz), 134.9 (d, *J* = 9.9 Hz), 131.7 (d, *J* = 12.4 Hz), 123.0 (d, *J* = 7.7 Hz), 120.1 (d, *J* = 86.4 Hz), 113.0 (d, *J* = 23.4 Hz), 111.1, 108.1 (d, *J* = 2.1 Hz), 48.2, 44.8, 40.0, 37.0, 31.1 (d, *J* = 16.3 Hz), 30.1, 27.2, 23.4 (d, *J* = 4.2 Hz), 22.7 (d, *J* = 50.9 Hz) and 8.8 ppm; ESI-MS (*m*/*z*): $[M]^+$ calcd for $C_{41}H_{45}FN_4O_2P$: 675.33; found: 675.35

Hemolysis Assay

The hemolysis assay was performed using the mouse red blood cell (RBC) to confirm whether the CFX and CFX-PPh3 derivatives inhibit the *S. aureus* induced hemolytic activity [S4]. RBCs was

collected from 7-weeks male C57BL/6J mouse. Firstly, *S. aureus* (MSSA ATCC29213) were cultured in 3 mL of CA-MHB media with 1/8× MIC, 1/4× MIC, 1/2× MIC and 1× MIC of CFX and CFX-derivatives. After overnight incubation, the bacteria culture of 3 mL was collected by centrifugation at 3000 rpm for 5 min at 4 °C and washed with cold PBS (pH 7.4) three times. The bacteria pellets were re-suspended in 500 μL of cold PBS (pH 7.4). RBCs were washed with cold phosphate-buffered saline (PBS, pH 7.4, 4 °C) three times by centrifugation (2000 g for 10 min) and then diluted in PBS to a final concentration of 8%. A 50 μL of bacteria solution was resuspended in the 450 μL of 8% suspension of RBC (1:9 ratio) in e-tube and incubated at 37 °C for 1 h. The PBS was used as a negative control, and the Triton X-100 (0.1% *v/v*, Sigma-Aldrich, Product No. 9002-93-1, USA) was used as a positive control. After 1 h incubation, the supernatants were collected by centrifugation (2000 g for 10 min) and then transferred to a 96-well plate. The absorbance of the solution was measured at 540 nm using a TECAN microplate reader (Spark 10M, Tecan, Craishim, Germany). The percentage of hemolysis was calculated by the following equation:

$$\% \text{ Hemolysis} = (\text{Abs}_{\text{sample}} - \text{Abs}_{\text{negative control}})/(\text{Abs}_{\text{positive control}} - \text{Abs}_{\text{negative control}}) \times 100$$

Membrane depolarization assay

The membrane depolarization activities of the CFX and CFX-PPh_3 against MSSA (ATCC29213) and MRSA derivatives was performed by using membrane potential-sensitive fluorescent dye, 3,3`-dipropylthiadicarbocyanine iodide ($DiSC_3$; Sigma-Aldrich, Product No. 53213-94-8, USA), as previously described by Shuai-Cheng W et al. [S5]. The MSSA and MRSA were cultured in the CA-MHB for 20 h and washed three times with PBS (pH 7.4) containing 20 mM glucose (PBS-glu). The washed bacteria suspension was adjusted to a 0.5 McFarland turbidity standard (1×10^8 CFU/mL) in PBS-glu. Aliquots 50 μL of adjusted bacteria suspension was added in 96-well black flat bottom plate, and then incubated with 5 μM $DiSC_3$ at 37 °C for 30 min. After that, the MSSA and MRSA were incubated with CFX and CFX-PPh3 at different concentrations (0.5×, 1.0×, 2.0× and 4.0× MIC) for 80 min, and the fluorescence intensity of $DiSC_3$ was monitored every 5 min for 80 min using a TECAN microplate reader (Spark 10M, Tecan, Craishim, Germany) at an excitation wavelength of 622 nm and an emission wavelength of 670 nm.

2. Supporting Figures

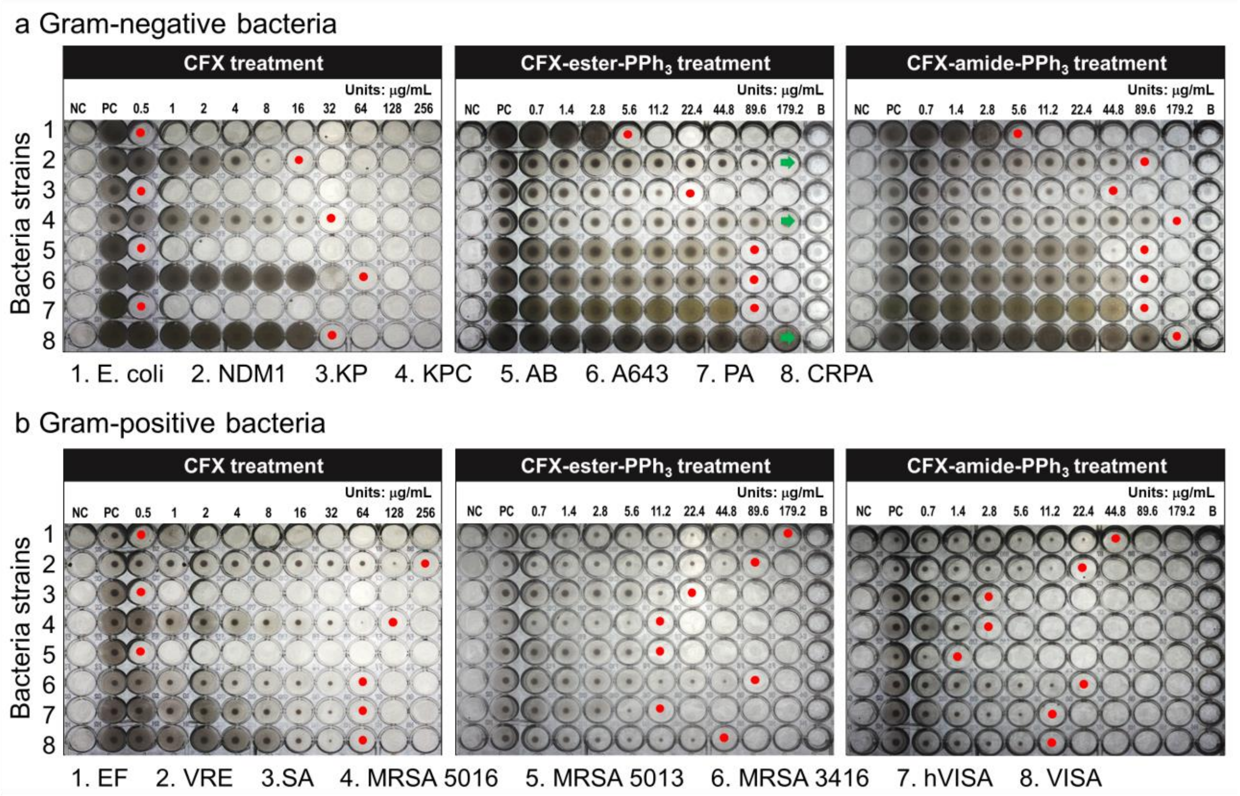

Figure 1. Photos of MIC results of CFX and PPh_3 modified CFXs against the 16 types of MDR bacteria using a 96-well microplate. MIC assay was performed using the following CLSI guidelines. Gram-negative; 4 types (*E. coli*, *K. pneumoniae*, *A. bacumannii* and *P. aeruginosa*) and Gram-positive; 2 types (*S. aureus* and *E. faecium*) bacteria were used as quality control strains. CFX and CFX- PPh_3 derivatives were serially diluted from column 3 to 12 of a 96-well microplate with concentration gradients (concentration range: CFX for 512–0.00005 µg/mL, CFX-PPh_3 derivatives for 178.5–1.39 µg/mL). CA-MHB (absent inoculum) was used as a negative control (column 1), and inoculated strains were used as positive controls (column 2). Red dot: MIC values. Green arrow: growth over.

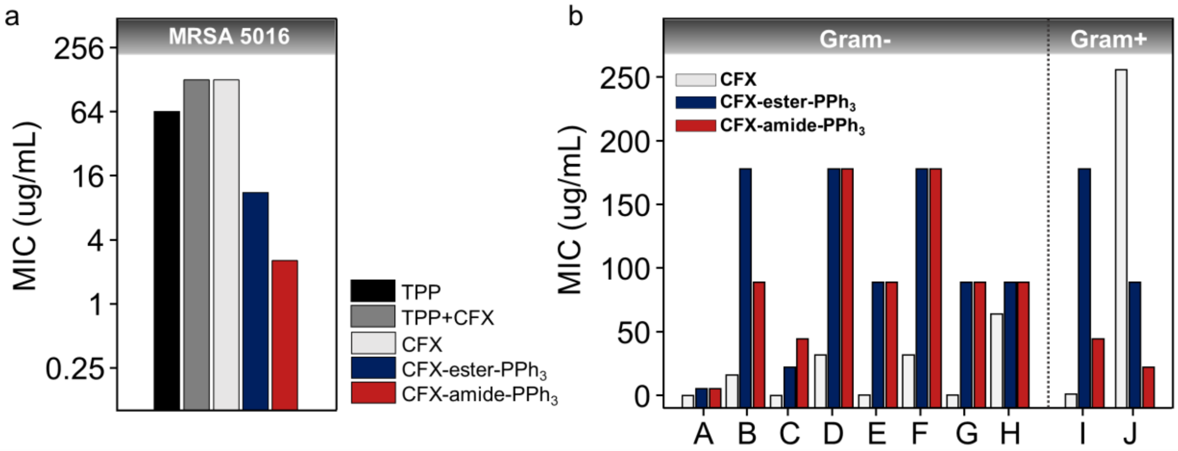

Figure 2. Screening results of Minimum inhibitory concentration (MIC) of CFX, CFX-ester-PPh_3, and CFX-amide-PPh_3 against ESKAPE pathogens of the total 10 types, including the MDR bacteria (a) The MIC assay for TPP^+ (Heptyltriphenylphosphonium Bromide) against MRSA 5016 strain. The experimental conditions are i) only TPP^+, ii) only CFX, iii) blended TPP^+ and CFX, iv) CFX-ester-PPh_3, and v) CFX-amide-PPh_3. (b) A-H indicated the results of Gram-negative bacteria; A: *E. coli*, B: *E. coli* (NDM-1 type), C: *K. pneumoniae*, D: *K. pneumoniae* (KPC type), E: *A. bacumannii* F: Carbapenem-resistant *A. baumannii* (CRAB), G: *P. aeruginosa,* H: Carbapenem-resistant *P. aeruginosa* (CRPA). I and J indicated the results of Gram-positive bacteria; I: *E. faecium* and J: Vancomycin-resistant *E. faecium* (VRE).

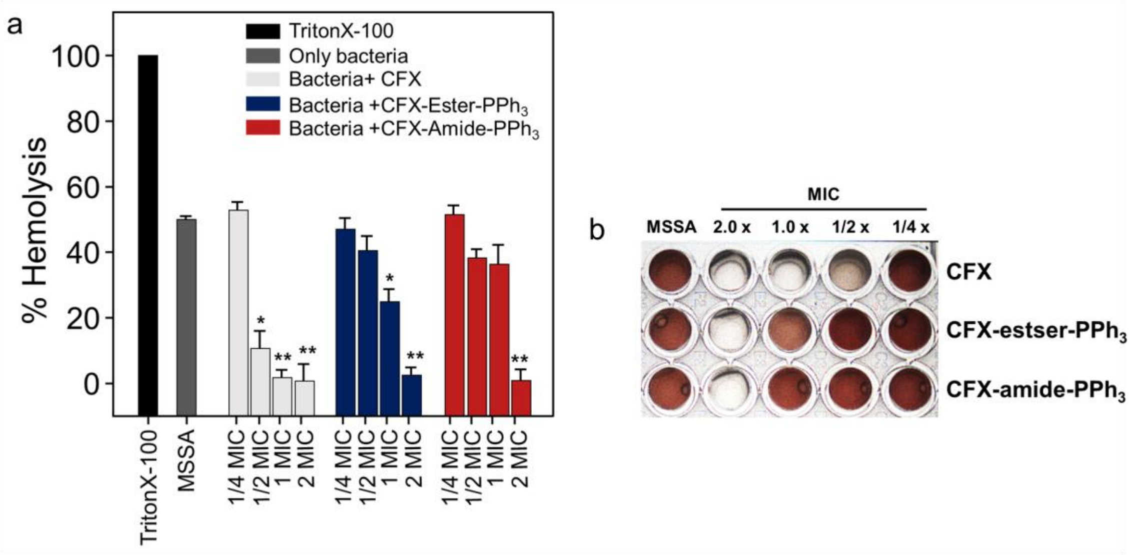

Figure 3. Effect of CFX and CFX-PPh_3 derivatives on mouse RBC lysis induced by MSSA. (a) shows hemolysis rate (%) compared to a positive control (Triton-X 100). (b) shows a picture of 96 well plate for measuring the absorbance (OD 540nm). Mouse RBC were incubated with MSSA with different MIC of CFX and CFX-PPh_3 derivatives. TritonX-100 (0.1%) and PBS were used as a positive control and negative control, respectively. Bars represents mean ± SD from three independent experiment. .

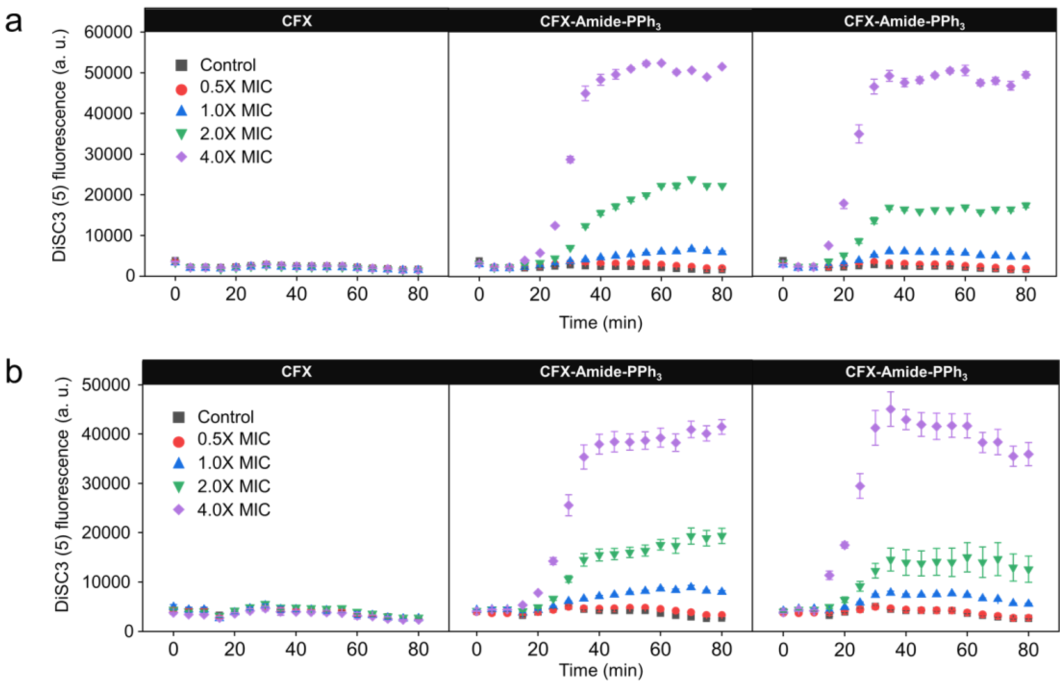

Figure 4. Time-dependent fluorescence intensity changes of $DiSC_3$ within the (a) MSSA and (b) MRSA 5016 after treatment of CFX and CFX-PPh_3 derivatives. An excitation wavelength of 622 nm and an emission wavelength of 670 nm was used for the intensity monitoring. Each data point represents the mean ± SD from three independent experiments.

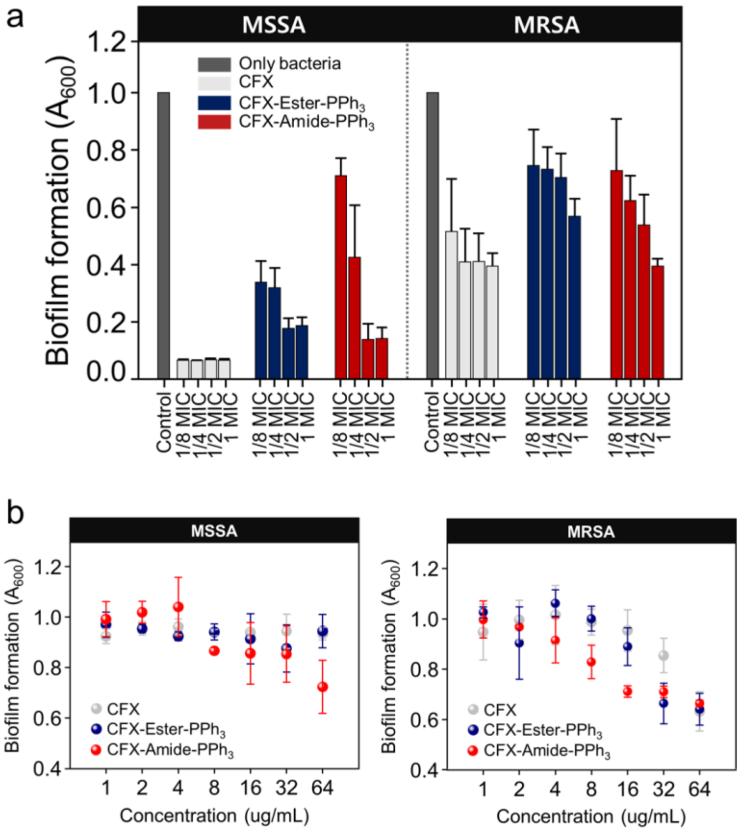

Figure 5. Antibiofilm activity (inhibition of biofilm formation) against MSSA and MRSA 5016 treated with CFX, CFX-PPh$_3$ derivatives at sub MIC. (a) Inhibitory activity of biofilm formation at sub-MIC of CFX, CFX-ester-PPh$_3$, and CFX-amide-PPh$_3$ against MSSA and MRSA 5016. (b) Ability to disrupt the preformed bacterial biofilm by CFX and CFX-PPh$_3$ derivatives. Biofilm formed by MSSA and MRSA 5016 were stained with crystal violet for 10 min and eluted in 33% acetic acid. The biofilm mass was measured at 600 nm of optical density (OD) values.

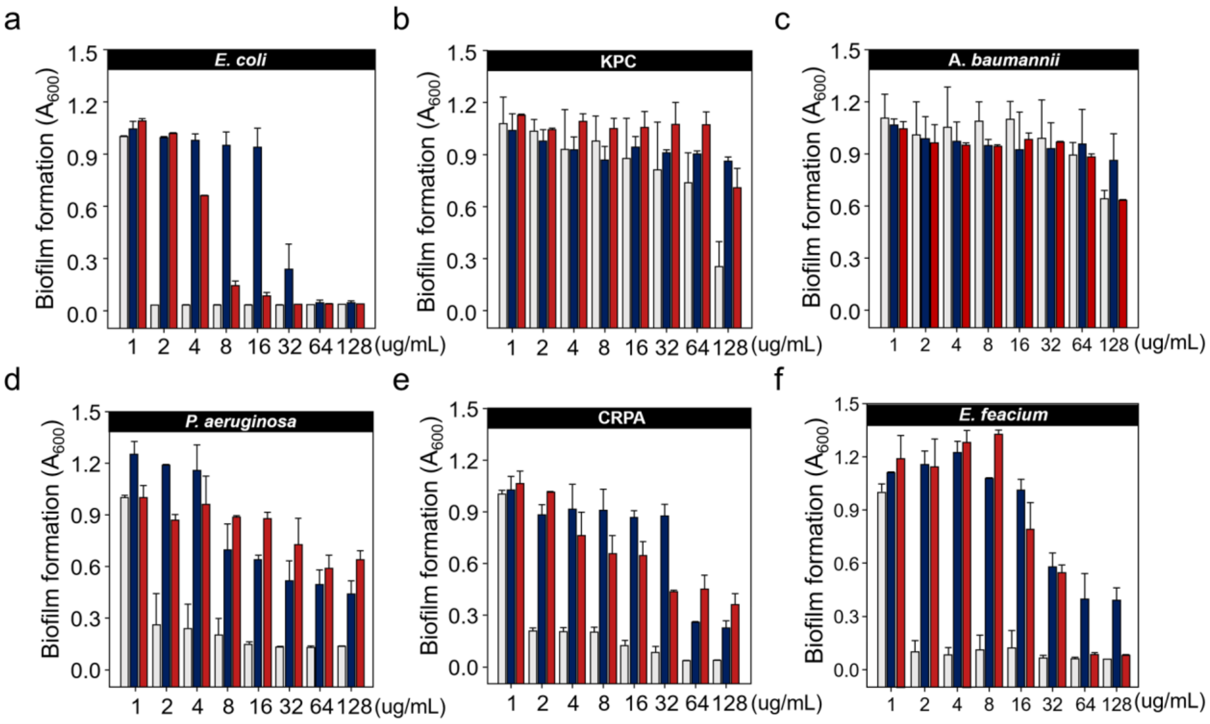

Figure 6. Antibiofilm activity (inhibition of biofilm formation) against ESKAPE treated with CFX, CFX-ester-PPh3 and CFX-ester-PPh3 at different concentrations (n=3). Gram-negative; 4 types (*E. coli*, *K. pneumoniae*, *A. bacumannii* and *P. aeruginosa*) and gram-positive; 2 types (*S. aureus* and *E. faecium*) bacteria were used as quality control strains. The biofilm mass was stained with crystal violet and the optical density was measured at 600 nm. Data are presented as the mean ± standard deviation from three independent experiments.

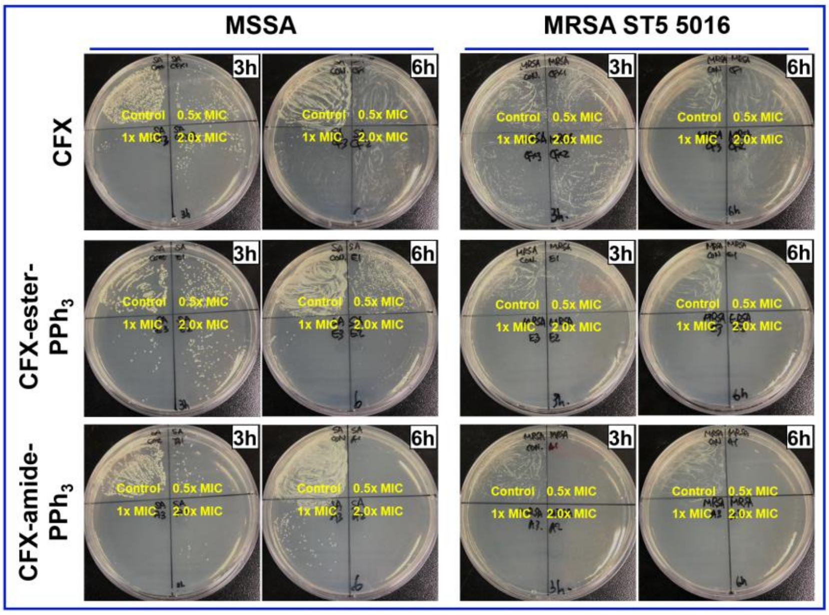

Figure 7. Photos of agar plates, MSSA and MRSA colonies (CFU/mL) grown on LB agar palate after treatment of CFX and CFX-PPh_3 derivatives. MSSA and MRSA were treated with CFX and CFX-PPh_3 derivatives at 0.5× , 1×, 2× MIC concentration of each compound. After exposures of 3 h and 6 h of the CFX and CFX derivates to MSSA and MRSA, they were then diluted and spread on LB plate. After incubating for 24 h, the viable bacteria cells were determined by counting CFU (colony forming unit) and multiplying the results by dilution factors. The photos of MSSA and MRSA CFU (early inoculum; approximately 10^6 CFU/mL) grown for 24 h in the LB plate are indicated in the graph (Figure 3a).

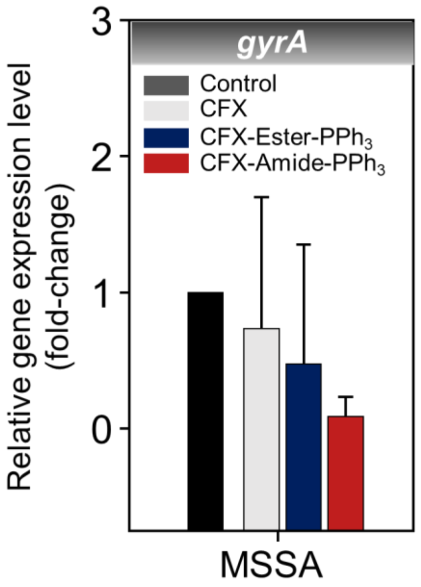

Figure 8. Gene expression level of *gyrA* in MSSA after treatment of CFX and CFX derivatives. The gene expression level was measured after CFX and CFX-PPh_3 derivative treatment at 0.5× MIC in comparison to the drug-free growth used as control. Each *Ct* (cycle threshold) value was normalized to 16S rRNA as internal control, and the normalized fold change was calculated using the delta-delta *Ct* method, with a drug-free group being the control. The results are shown as the means ± standard deviation of triplicate independent experiments.

3. Supporting Tables

Table 1. Primer sequences used in this study.

Genes	Primer Sequences (5`-3`)	Ref.
norA	F: 5'-GCA GTC GAG CAT TTA ATG GA-3'<br>R: 5'-ACG TTG TTG CAA CTG TGT AAG A-3'	T1
sepA	F: 5'-GCA GTC GAG CAT TTA ATG GA-3'<br>R: 5'-ACG TTG TTG CAA CTG TGT AAG A-3'	T 2
mdeA	F: 5'-AAC GCG ATA CCA ACC ATT C-3'<br>R: 5'-TTA GCA CCA GCT ATT GGA CCT-3'	T 2
16S rRNA	F: 5'-GAC GCT CGA ACC AAT GGT ATT GC-3'<br>R: 5'-GCC AGT TGG AAA ATC AGG ACC TT-3'	T 2

Table 2. Biofilm formation Inhibition rate at ½ sub-MIC concentration of CFX and CFX-PPh_3 derivatives.

	**CFX**	**CFX-Ester-PPh3**	**CFX-Amide-PPh3**
MSSA	93.2%	82.3%	86.1%
MRSA ST5 5016	58.9%	26.7%	46.1%

4. Supporting References

S1. C. Lee, H.-K. Park, H. Jeong, J. Lim, A.-J. Lee, K. Y. Cheon, C.-S. Kim, A. P. Thomas, B. Bae and N. D. Kim. *Journal of the American Chemical Society*. **2015**;137:4358-4367.

S2. J. Chen, X. Jiang, C. Zhang, K. R. MacKenzie, F. Stossi, T. Palzkill, M. C. Wang and J. Wang. *ACS sensors*. **2017**;2:1257-1261.

S3. K. S. Tanaka, T. J. Houghton, T. Kang, E. Dietrich, D. Delorme, S. S. Ferreira, L. Caron, F. Viens, F.
F. Arhin and I. Sarmiento. *Bioorganic & medicinal chemistry*. **2008**;16:9217-9229.

S4. S. Lin, J.-J. Koh, T. T. Aung, W. L. W. Sin, F. Lim, L. Wang, R. Lakshminarayanan, L. Zhou, D. T. Tan and D. Cao. *Journal of Medicinal Chemistry*. **2017**;60:6152-6165.

S5. W. Shuai-cheng, L. Qian, Y. Zhi-Qiang, L. Fei, P. Wen-Jing, S. Xiang-Bin, Q. Shao-Qi, K. Zhu and S. JIan-Zhong. *Frontiers in Microbiology*. **2019**;10:2489.

T1. A. Salehzadeh, M. S. Hashemi Doulabi, B. Sohrabnia and A. Jalali. *Journal of Genetic Resources*. **2018**;4:26-36.

T2. S. Hassanzadeh, R. Mashhadi, M. Yousefi, E. Askari, M. Saniei and M. R. Pourmand. *Microbial pathogenesis*. **2017**;111:71-74.
